# Infectivity and fatality of influenza in pre- and post-COVID-19 pandemic year

**DOI:** 10.1371/journal.pcbi.1013229

**Published:** 2025-07-08

**Authors:** Shuanglin Jing, Hao Wang

**Affiliations:** 1 School of Mathematics and Physics, Lanzhou Jiaotong University, Lanzhou, Gansu, China; 2 Gansu Center for Fundamental Research in Complex Systems Analysis and Control, Lanzhou Jiaotong University, Lanzhou, Gansu, China; 3 Department of Mathematical and Statistical Sciences, University of Alberta, Edmonton, Alberta, Canada; Shanxi University, CHINA

## Abstract

The COVID-19 pandemic and related non-pharmaceutical interventions (NPIs) significantly alter the transmission dynamics of non-SARS-CoV-2 infectious diseases, with respiratory infections such as influenza being disproportionately affected. We aim to compare influenza’s epidemiological characteristics between pre-pandemic and post-pandemic periods to inform public health responses. We develop two influenza transmission models incorporating age structure and multi-strain dynamics, featuring time-varying transmission and mortality rates. Using publicly available U.S. data, we calibrate these models to evaluate age- and strain-specific transmission patterns and mortality rates across different pandemic eras. Our analysis reveals that during the final pandemic year, influenza transmission among adults (≥18 years) initially declined but rebounded to pre-pandemic levels within the first post-pandemic year following NPI relaxation and behavioral normalization, while transmission stability persists in the <18 cohort. All-age influenza mortality rates exhibit a transient elevation during the pandemic’s final year before returning to baseline levels pre-pandemic. Furthermore, after the COVID-19 pandemic, the transmission rate of influenza A decreases alongside peaks in new cases, while the transmission of influenza B fluctuates without a decline. Our findings indicate that while the COVID-19 pandemic has induced significant transient modifications in influenza’s epidemiological profile, key transmission and mortality characteristics regain pre-pandemic equilibrium within one year following pandemic resolution.

## Author summary

This study examines how COVID-19 pandemic-era interventions influence influenza transmission and mortality dynamics in the U.S. Using age-structured and multi-strain models calibrated to pre-pandemic (2016–2019), pandemic (2022–2023), and post-pandemic (2023–2024) data, we find that adult influenza transmission rates temporarily decline during the pandemic but rebound to pre-pandemic levels within a year of NPI relaxation, while pediatric transmission remains stable. All-age mortality rates rise transiently during the pandemic, likely due to healthcare disruptions, before returning to baseline post-pandemic. Influenza A transmission decreases during the pandemic but recovers afterward, whereas influenza B exhibits fluctuating trends. These findings highlight the transient impact of pandemic-related behavioral and systemic changes on influenza epidemiology, emphasizing the resilience of pre-pandemic transmission equilibria and the need for strain- and age-specific public health strategies.

## Introduction

The emergence of the COVID-19 pandemic has profoundly transformed the global landscape of infectious disease management, presenting unprecedented challenges to healthcare systems worldwide. In response to this crisis, governments implemented rigorous containment strategies including travel restrictions, enforced social distancing protocols, and universal mask mandates [1,2]. These interventions not only effectively mitigated SARS-CoV-2 transmission but also substantially reduced the incidence of various other infectious diseases, including influenza, respiratory syncytial virus infections, malaria, syphilis, and brucellosis [3–7]. Epidemiological surveillance data revealed significant declines in both positivity rates and case numbers for these conditions during pandemic years [3,4,7].

While numerous studies have examined COVID-19’s influence on infectious disease epidemiology, existing research predominantly focuses on transmission dynamics during pandemic restrictions [3–5,8] or immediate post-pandemic periods [7,9], with limited investigation into comparative analyses of pre- and post-pandemic transmission patterns. Furthermore, current literature emphasizes incidence rate fluctuations [3–5,7,8,10], while quantitative assessments of transmission efficiency and mortality rates remain understudied.

As a seasonal respiratory pathogen, influenza epidemiology demonstrates particular sensitivity to non-pharmaceutical interventions (NPIs). Prior to the pandemic, influenza exhibited predictable transmission patterns with well-characterized population-level impacts [11,12]. The COVID-19 crisis precipitated fundamental shifts in healthcare prioritization, public health infrastructure, and health-seeking behaviors, potentially altering influenza’s epidemiological characteristics [13–15]. Infection control measures such as universal masking and social distancing likely contributed to observed reductions in influenza transmission [3]. However, the post-pandemic era raises critical questions regarding potential alterations in influenza’s transmission dynamics and virulence patterns, particularly concerning temporal variations in core epidemiological indicators.

To address these knowledge gaps, we developed two mathematical models informed by Kermack-McKendrick theory [16–20]: 1) an age-stratified transmission model examining demographic variations in disease progression, and 2) a multi-strain model for analyzing the competitive dynamics of different strains. These frameworks enable systematic investigation of two critical dimensions: first, the age-structured model facilitates analysis of heterogeneous transmission risks and mortality patterns across demographic groups; second, the multi-strain model permits simulation of competitive transmission dynamics and mortality variations among co-circulating influenza variants.

Model calibration utilized age-specific and strain-specific incidence and mortality data from U.S. influenza surveillance systems. Subsequent computational analyses yielded strain-specific and age-stratified estimates of infectivity, case fatality rates, and effective reproduction numbers. Our models demonstrated strong concordance with empirical epidemic curves, validating their capacity to capture influenza transmission dynamics. This study provides comprehensive quantitative analysis of COVID-19’s enduring impacts on influenza epidemiology, with particular focus on temporal variations in transmission efficiency and mortality outcomes across pandemic phases.

## Methods

In this section, we use two mathematical models to estimate the transmission and death rates of influenza. The first model is an age-structured model that divides the population into multiple age groups, with each group further segmented into susceptible, latent, infected, recovered, and deceased subclasses. This model captures the dynamics of influenza transmission across different age groups, taking into account factors such as natural mortality, age-group transition, and time-varying transmission and death rates. The second model is a multi-strain model that considers the transmission and death rates of multiple influenza strains within the entire population. This model includes susceptible, latent (with specific strain), infected (with specific strain), recovered, and deceased classes, allowing for the estimation of strain-specific transmission and death rates over time. Both models incorporate time-dependent parameters to reflect the variability in influenza transmission and severity.

### Age-structured model

To estimate the transmission and death rates of influenza infection across different age groups, we divide the total population into *n* age groups. Each age group is further subdivided into five subcategories: susceptible individuals (*S*_*k*_), latent individuals (*E*_*k*_), infected individuals (*I*_*k*_), recovered individuals (*R*_*k*_), and deceased individuals (*D*_*k*_). The population size of the *k*-th age group at time *t* is denoted as The force of infection among individuals in age-group *k* is defined as


$$N_{k}(t)=S_{k}(t)+E_{k}(t)+I_{k}(t)+R_{k}(t).$$

The overall population, *N*(*t*), is obtained by summing *N*_*k*_(*t*) over all age groups and grows at a rate of Λ. The population *N*_*k*_(*t*) of the *k*-th age group experiences natural mortality at a rate of $d_{k}N_{k}(t)$ and transitions to the next age group at a rate of $\alpha_{k}N_{k}(t)$, where *d*_*k*_ represents the natural mortality rate and $\alpha_{k}$ denotes the rate of transfer from age-group *k* to age-group *k* + 1.

The force of infection among individuals in age-group *k* is defined as


$$\sum_{j=1}^{n}\frac{\beta_{kj}(t)I_{j}(t)}{N_{j}(t)},\;\text{for }1\leq k\leq n,$$

where $\beta_{kj}(t)$ represents the transmission rate between individuals in age-group *k* and those in age-group *j*, and $\frac{I_{j}}{N_{j}}$ indicates the probability of encountering an infectious individual from age-group *j*. For the *k*-th age group, susceptible individuals become infected with the influenza virus and move to the latent class at a rate $S_{k}(t)\sum_{j=1}^{n}\frac{\beta_{kj}(t)I_{j}(t)}{N_{j}(t)}$. Latent individuals progress to the infected class at a rate $\sigma_{k}E_{k}(t)$, where $\frac{1}{\sigma_{k}}$ represents the duration of the latent period. Infected individuals transition to the recovered and deceased classes at rates $\left(1-\mu_{k}(t))\gamma_{k}I_{k}(t\right)$ and $\mu_{k}(t)\gamma_{k}I_{k}(t)$ respectively, with $\frac{1}{\gamma_{k}}$ representing the duration of the infectious period and $\mu_{k}(t)$ signifying the probability of death due to influenza. Recovered individuals, though not fully immune, revert to the susceptible class at a rate $\delta_{k}R_{k}(t)$, where $\delta_{k}$ represents the rate of immunity loss. Notably, both $\mu_{k}(t)$ and $\beta_{kj}(t)$ are time-varying parameters. A comprehensive list of these parameters is provided in Table A of S1 Text.

Our age-structured model is given by


$$\frac{\text{d}S_{1}(t)}{\text{d}t}=\Lambda -S_{1}(t)\sum_{j=1}^{n}\frac{\beta_{1j}(t)I_{j}(t)}{N_{j}(t)}-d_{1}S_{1}(t)-\alpha_{1}S_{1}(t)+\delta_{1}R_{1}(t),$$

$$\frac{\text{d}S_{k}(t)}{\text{d}t}=\alpha_{k-1}S_{k-1}(t)-S_{k}(t)\sum_{j=1}^{n}\frac{\beta_{kj}(t)I_{j}(t)}{N_{j}(t)}-d_{k}S_{k}(t)-\alpha_{k}S_{k}(t)+\delta_{k}R_{k}(t),\;2\leq k\leq n,$$

$$\frac{\text{d}E_{1}(t)}{\text{d}t}=S_{1}(t)\sum_{j=1}^{n}\frac{\beta_{1j}(t)I_{j}(t)}{N_{j}(t)}-\sigma_{1}E_{1}(t)-d_{1}E_{1}(t)-\alpha_{1}E_{1}(t),$$

$$\frac{\text{d}E_{k}(t)}{\text{d}t}=\alpha_{k-1}E_{k-1}(t)+S_{k}(t)\sum_{j=1}^{n}\frac{\beta_{kj}(t)I_{j}(t)}{N_{j}(t)}-\sigma_{k}E_{k}(t)-d_{k}E_{k}(t)-\alpha_{k}E_{k}(t),\;\;2\leq k\leq n,$$

$$\frac{\text{d}I_{1}(t)}{\text{d}t}=\sigma_{1}E_{1}(t)-\gamma_{1}I_{1}(t)-d_{1}I_{1}(t)-\alpha_{1}I_{1}(t),$$

$$\frac{\text{d}I_{k}(t)}{\text{d}t}=\alpha_{k-1}I_{k-1}(t)+\sigma_{k}E_{k}(t)-\gamma_{k}I_{k}(t)-d_{k}I_{k}(t)-\alpha_{k}I_{k}(t),\;\;2\leq k\leq n,$$ (1)

$$\frac{\text{d}R_{1}(t)}{\text{d}t}=(1-\mu_{1}(t))\gamma_{1}I_{1}(t)-d_{1}R_{1}(t)-\alpha_{1}R_{1}(t)-\delta_{1}R_{1}(t),$$

$$\frac{\text{d}R_{k}(t)}{\text{d}t}=\alpha_{k-1}R_{k-1}(t)+(1-\mu_{k}(t))\gamma_{k}I_{k}(t)-d_{k}R_{k}(t)-\alpha_{k}R_{k}(t)-\delta_{k}R_{k}(t),\;\;2\leq k\leq n,$$

$$\frac{\text{d}D_{1}(t)}{\text{d}t}=\mu_{1}(t)\gamma_{1}I_{1}(t),$$

$$\frac{\text{d}D_{k}(t)}{\text{d}t}=\mu_{k}(t)\gamma_{k}I_{k}(t),\;\;2\leq k\leq n.$$

According to the Model (1), the formulas for the weekly number of new cases and new deaths in the *k*-th age group are


$$C_{k}^{AS}(j)=\int_{\text{week}\;j}\sigma_{k}E_{k}(t)\text{d}t,\;\;1\leq k\leq n$$

and


$$U_{k}^{AS}(j)=\int_{\text{week}\;j}\mu_{k}(t)\gamma_{k}I_{k}(t)\text{d}t,\;\;1\leq k\leq n,$$

respectively.

The basic reproduction number, ${ℛ}_{0}^{AS}$, is calculated using the next generation matrix approach as outlined by van den Driessche and Watmough [26]. However, considering the variability of the transmission rate over time, the effective reproduction number, ${ℛ}_{e}^{AS}(t)$, is a more dynamic measure. The expression for ${ℛ}_{e}^{AS}(t)$ is

$${ℛ}_{e}^{AS}(t)=\rho (F_{AS}(t)V_{AS}^{-1}),$$ (2)

where it is defined in terms of the time-dependent transmission rate matrix $F_{AS}(t)$ and the inverse of the transition matrix ${𝒱}_{AS}$ (see S1 Text).

### Multi-strain model

To estimate the transmission and death rates of influenza by strain, we construct a mathematical model in which both the transmission and death rates vary over time. The whole population is divided into five classes, namely *S*(*t*), *E*_*i*_(*t*), *I*_*i*_(*t*), *R*(*t*), and *D*(*t*). These respectively represent the numbers of individuals who are: (i) susceptible; (ii) latent with infection strain *i*; (iii) infectious with infection strain *i*; (iv) recovered; and (v) dead due to the infection. The total population is denoted by


$$N(t)=S(t)+\sum_{i=1}^{m}E_{i}(t)+\sum_{i=1}^{m}I_{i}(t)+R(t).$$

Similar to Model (1), Λ and *d* stand for the recruitment rate and the natural mortality rate of the population, respectively. δ represents the rate at which a recovered individual loses immunity. $1/\sigma_{i}$ denotes the length of the latent period for individuals infected with strain *i*. Furthermore, we denote by $\beta_{i}(t)$, $\gamma_{i}$, $\mu_{i}(t)$ the per capita transmission rate, recovery rate, and probability of death, respectively, corresponding to individuals infected with strain *i*. As in Model (1), we assume that $\mu_{i}(t)$ and $\beta_{i}(t)$ are time-dependent. A comprehensive list of these parameters is provided in Table B of S1 Text.

Our multi-strain model is given by


$$\frac{\text{d}S(t)}{\text{d}t}=\Lambda -S(t)\sum_{i=1}^{m}\frac{\beta_{i}(t)I_{i}(t)}{N(t)}-dS(t)+\delta R(t),$$

$$\frac{\text{d}E_{i}(t)}{\text{d}t}=S(t)\frac{\beta_{i}(t)I_{i}(t)}{N(t)}-\sigma_{i}E_{i}(t)-dE_{i}(t),\;1\leq i\leq m,$$

$$\frac{\text{d}I_{i}(t)}{\text{d}t}=\sigma_{i}E_{i}(t)-\gamma_{i}I_{i}(t)-dI_{i}(t),\;\;\;\;\;\;1\leq i\leq m,$$ (3)

$$\frac{\text{d}R(t)}{\text{d}t}=\sum_{i=1}^{m}(1-\mu_{i}(t))\gamma_{i}I_{i}(t)-dR(t)-\delta R(t),$$

$$\frac{\text{d}D(t)}{\text{d}t}=\sum_{i=1}^{m}\mu_{i}(t)\gamma_{i}I_{i}(t).$$

Similar to the Model (1), the formulas for the weekly number of new cases and new deaths of infected strain *i* are


$$C_{i}^{MS}(j)=\int_{\text{week}\;j}\sigma_{i}E_{i}(t)\text{d}t,\;\;1\leq i\leq m$$

and


$$U_{i}^{MS}(j)=\int_{\text{week}\;j}\mu_{i}(t)\gamma_{i}I_{i}(t)\text{d}t,\;\;1\leq i\leq m,$$

respectively.

The effective reproduction number of Model (3) is defined as

$${ℛ}_{e}^{MS}(t)=\rho (F_{MS}(t)V_{MS}^{-1}),$$ (4)

where the expressions for $F_{MS}(t)$ and $V_{MS}^{-1}$ can be found in S1 Text.

### Data collection

In order to simulate the impact of the COVID-19 pandemic on influenza in the United States, we collect laboratory-confirmed influenza hospitalization and influenza death data from the National Center for Health Statistics Mortality Surveillance System for weeks 40 to 17 of the periods 2016-2017, 2017-2018, 2018-2019, 2022-2023, and 2023-2024, respectively (see Fig A in S1 Text) [25,28]. Since the Influenza Hospitalization Surveillance Network (FluSurv-NET) conducts population-based surveillance for laboratory-confirmed influenza-associated hospitalizations among children (persons younger than 18 years) and adults. The current network covers over 90 counties or county equivalents in the 10 Emerging Infections Program states (CA, CO, CT, GA, MD, MN, NM, NY, OR, and TN) and four additional states through the Influenza Hospitalization Surveillance Project (MI, NC, OH, and UT). The network represents approximately 9% of the US population (≈ 30 million people) [25]. Hence, we recalculate the total number of influenza hospitalization cases according to the total US population of 330 million. In addition, we also collect weekly confirmed cases of influenza by strain from sentinel and non-sentinel surveillance (see Fig B in S1 Text) [27]. Note that we do not have access to the number of influenza deaths categorized by strain.

### Parameter estimation

In our simulations, we categorize the population of Model (1) into three distinct age groups: 0–17 years old, 18–64 years old, and those over 65 years old. We obtain the total population and the population figures for each age group from World Health Organization (WHO) data for the country under study [21]. Additionally, we calculate the initial values for the model using influenza vaccination rates across these age groups, as reported by the Centers for Disease Control and Prevention (CDC) [24]. With the aim of aligning with the temporal resolution of the observed epidemiological data, we set the time step in our simulations to one week. Subsequently, we proceed to estimate all parameters and initial values for Model (1).

(I) The initial values of Model (1): In the simulated 5-year period, we assume that the initial values of susceptible individuals in the three age groups are $S_{1}(0)=0.55N_{1}$, $S_{2}(0)=0.5N_{2}$, and $S_{3}(0)=0.3N_{3}$, respectively, where *N*_1_, *N*_2_, and *N*_3_ represent the total population of the three age groups (see Table A in S1 Text). Similarly, the initial values of recovered individuals in the three age groups are $R_{1}(0)=0.45N_{1}$, $R_{2}(0)=0.5N_{2}$, and $R_{3}(0)=0.7N_{3}$, respectively, where constants 0.45, 0.5, and 0.7 represent the vaccination rates of the three age groups [24]. We also assume that the initial numbers of infected individuals in the *k*-th age group is $I_{k}(0)=\sigma_{k}E_{k}(0)$, and the initial number of infected individuals is assumed to be the same as the number of initially infected individuals, which means that the initial number of latent individuals can be obtained.

(II) The birth rate of the population (i.e. Λ): According to the statistics of the WHO [21], we assume that the number of births per week is 69230, i.e. Λ=69230.

(III) The natural mortality rate of the population (i.e. *d*_*k*_): According to the statistics of the WHO [21], we obtain that the average lifetime of Americans is 76 years, Thus, the weekly natural mortality rates are $d_{1}=1/\left(76\times 52\right)$, $d_{2}=1/\left(58\times 52\right)$, and $d_{3}=1/\left(12\times 52\right)$.

(IV) The rate of aging (i.e. $\alpha_{k}$): Given that the three age groups are categorized as 0-17 years, 18-64 years, and 65+ years, it implies that the population in the first age group transitions to the second age group after 18 years, and subsequently, after an additional 46 years, the population in the second age group transitions to the third age group, whereas the population in the third age group does not further transition but will only be subject to natural mortality. Therefore, we have


$$\alpha_{k}=\left\{ \begin{array}{cc} 1/(18\times 52), & k=1,\\ 1/(46\times 52), & k=2,\\ 0, & k=3. \end{array}\right.$$

(V) The latent period length of infected individuals (i.e. $1/\sigma_{k}$): According to the WHO’s detailed report on influenza [22], symptoms of infected individuals begin 1-4 days after infection and usually last around a week. Thus, we have $\sigma_{k}=7/2$.

(VI) The infectious period of infected individuals (i.e. $1/\gamma_{k}$): According to (V), $\gamma_{k}=1$.

(VII) The rate at which a recovered individual loses immunity (i.e. $\delta_{k}$): Influenza usually grants lifelong immunity to the specific strain that infects a person, with loss of immunity due to antigenic drift. Generally, infection in one year offers nearly full protection against current strains for one years, except for rare antigenic shifts [23]. Hence, we assume that $\delta_{k}=1/52$.

(VIII) The transmission rate between individuals in age-group *k* and individuals in age-group *j* (i.e. $\beta_{kj}(t)$): The transmission rate, $\beta_{kj}(t)(k,j=1,2,3)$, is assumed to be a piecewise cubic spline function with $n_{\beta }$ nodes [2,29], i.e. we let $\beta_{kj}(t)={{\text{PCSF}}_{\beta }}_{kj}(t)$.

(IX) The probability of deaths among infected individuals (i.e. $\mu_{k}(t)$): In order to estimate the time-varying mortality rate $\mu_{k}(t)(k=1,2,3)$, we let $C_{k}^{AS}(t)$ and $U_{k}^{AS}(t)$ be the weekly number of new hospitalizations and deaths in the *k*-th age group, respectively. Next, we apply spline functions to interpolate the observed data and generate smooth curves representing $C_{k}^{AS}(t)$ and $U_{k}^{AS}(t)$ [30,31]. In keeping with Model (1), we use the values of $\sigma_{k}E_{k}(t)$ and $\mu_{k}(t)\gamma_{k}I_{k}(t)$ as approximations of the weekly number of new hospitalizations and deaths in the *k*-th age group, respectively; in other words, we let $C_{k}^{AS}(t)=\sigma_{k}E_{k}(t)$ and $U_{k}^{AS}(t)=\mu_{k}(t)\gamma_{k}I_{k}(t)$. Since $C_{k}^{AS}(t)$ and $U_{k}^{AS}(t)$ are known, we obtain


$$E_{k}(t)=\frac{C_{k}^{AS}(t)}{\sigma_{k}},\;k=1,2,3.$$

According to Model (1), we have


$$\left\{ \begin{array}{c} \frac{\text{d}I_{1}(t)}{\text{d}t}=C_{1}^{AS}(t)-\gamma_{1}I_{1}(t)-d_{1}I_{1}(t)-\alpha_{1}I_{1}(t),\\ \frac{\text{d}I_{k}(t)}{\text{d}t}=\alpha_{k-1}I_{k-1}(t)+C_{k}^{AS}(t)-\gamma_{k}I_{k}(t)-d_{k}I_{k}(t)-\alpha_{k}I_{k}(t),\;k=2,3. \end{array}\right.$$

where *I*_*k*_(0) can be estimated as $C_{k}^{AS}(0)$, $C_{k}^{AS}(0)$ is the initial number of infected individuals, then, $C_{k}^{AS}(0)>0$. Therefore, the probability of death from influenza in the *k*-th age group is


$$\mu_{k}(t)=\frac{U_{k}^{AS}(t)}{\gamma_{k}I_{k}(t)},\;k=1,2,3.$$

We assume the observed data model to be


$$Y_{C_{k}}(t)=C_{k}^{AS}(t,\hat{\theta })+\varepsilon_{k},\;k=1,2,3,$$

where $Y_{C_{k}}(t)$ denotes the weekly number of new hospitalizations in the *k*-th age group. $\hat{\theta }$ denotes the unknown parameters of the model. The measurement errors, $\varepsilon_{k}$ is assumed to be independent Gaussian distributions with mean zero and unknown variances $\xi_{k}^{2}$. Next, we use the MCMC method [32] for 500000 iterations with a burn-in of 450000 iterations to fit Model (1). The MCMC objective functions for our model are as follows:


$$SS_{C_{k}}(\hat{\theta })=\overset{T}{\underset{t=0}{\sum }}[(Y_{C_{k}}(t)-C_{k}^{AS}(t,\hat{\theta }){)}^{2}],\;k=1,2,3,$$

where *T* is the end of the simulation.

Since the seasonal prevalence of influenza mainly caused by the transmission of influenza A and B viruses [22], for Model (3), we only divide influenza into types A and B strains in the simulation, and the parameter estimation process is consistent with Model (1) (see Table B in S1 Text).

## Results

**Fig 1:**
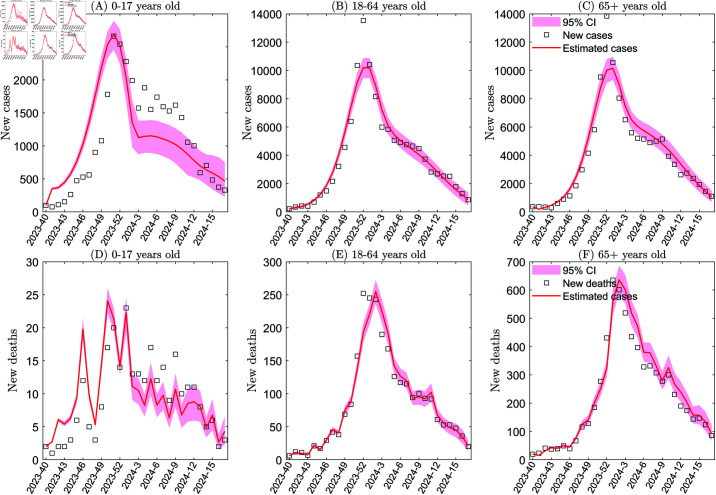
Age-structure model fitting based on data for influenza case and deaths from the 40th week of 2023 to the 17th week of 2024 for three age groups in the United States (i.e. 0–17 years old, 18–64 years old, and over 65 years old). Panels A, B, and C show the transmission rates fitting of influenza infected populations aged 0-17, 18-64, and 65+, respectively, based on the weekly number of influenza hospitalization surveillance cases. Panels D, E, and F show the fitting of the death rates (in the same order), based on weekly numbers of new deaths. The transmission rates were taken to be cubic spline functions, with numbers of nodes $n_{\beta }$ equal to three.

**Fig 2:**
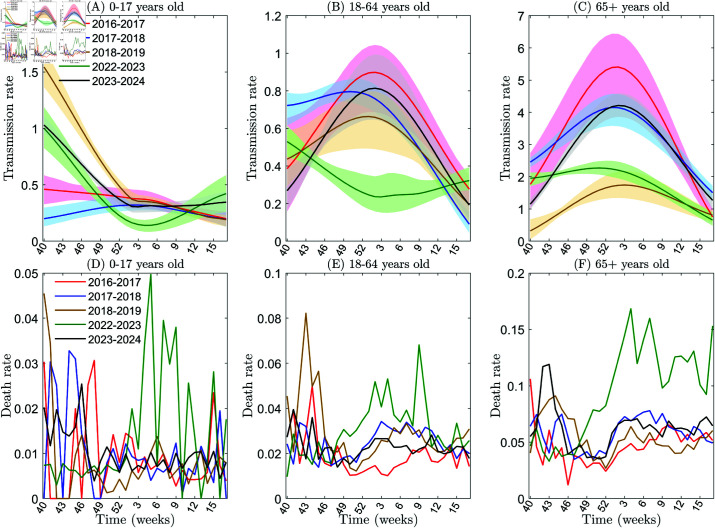
Estimation of influenza transmission and death rates among individuals aged 0–17, 18–64, and 65+ in the United States using actual data. Panels A, B, and C show the transmission rates of influenza among individuals aged 0–17, 18–64, and 65+, respectively. Panels D, E, and F show the death rates of influenza, in the same order. The 95% CI (confidence interval) for the simulations conducted in the years 2016–2017, 2017–2018, 2018–2019, 2022–2023, and 2023–2024 are plotted in pink, light blue, light yellow, light green, and gray, respectively.

**Fig 3:**
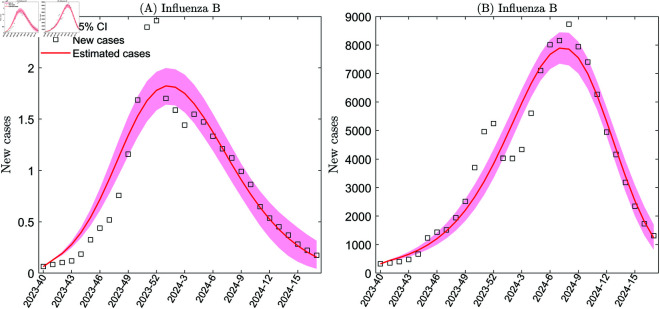
Multi-strain model fitting based on data for influenza A and B cases from the 40th week of 2023 to the 17th week of 2024 in the United States. Panels A and B show the transmission rates fitting of influenza A and B, respectively, based on the weekly number of influenza cases detected by sentinel and non-sentinel surveillance. The transmission rates were taken to be cubic spline functions, with numbers of nodes $n_{\beta }$ equal to three.

**Fig 4:**
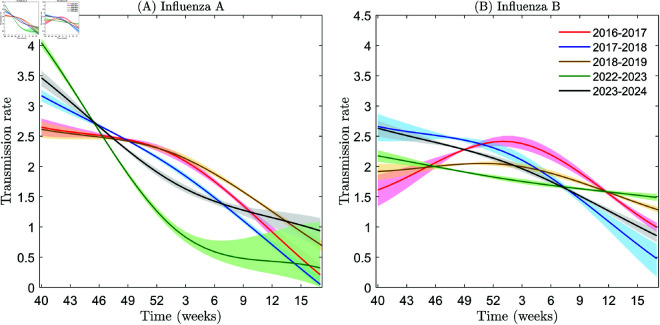
Estimation of transmission rates of influenza A and B in the United States using actual data. Panels A and B show the transmission rates of influenza A and B, respectively. The 95% CI for the simulations conducted in the years 2016–2017, 2017–2018, 2018–2019, 2022–2023, and 2023–2024 are plotted in pink, light blue, light yellow, light green, and gray, respectively.

**Fig 5:**
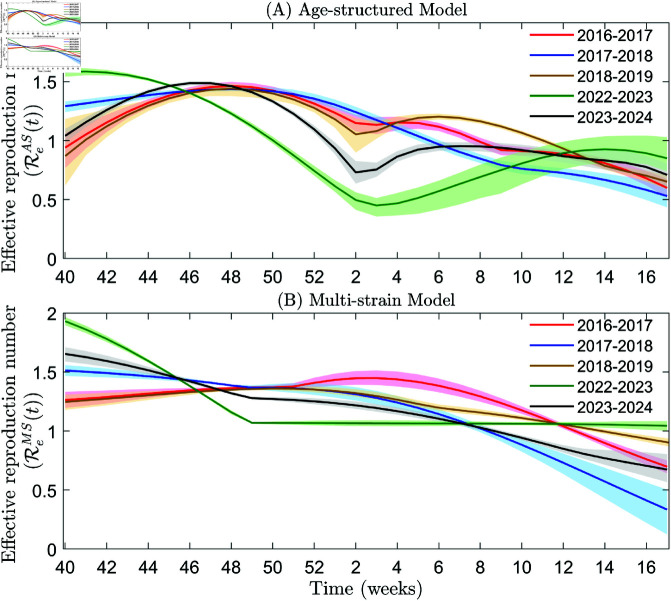
Effective reproduction numbers of influenza in the United States from week 40 to week 17 in 2016–2017, 2017–2018, 2018–2019, 2022–2023, and 2023–2024. Panels A and B show the effective reproduction numbers estimated using the age-structure model and the multi-strain model, respectively. The 95% CI for the simulations conducted in the years 2016–2017, 2017–2018, 2018–2019, 2022–2023, and 2023–2024 are plotted in pink, light blue, light yellow, light green, and gray, respectively.

In this section, we first estimate and compare the infectivity and fatality of influenza across different age groups in pre- and post-COVID-19 pandemic year, spanning specific weekly periods from 2016–2019 and 2022–2024 (as detailed in S1 Text and Figs 1 and 2). Subsequently, we use a multi-strain model to estimate the fatality of influenza A and B for the same time periods, presenting our findings in S1 Text and Figs 3 and 4. Finally, we calculate the effective reproduction numbers for both models using the estimated parameters, illustrating the results in Fig 5.

### Infectivity and fatality of influenza in different age groups

In this subsection, we estimate the transmission and death rates of influenza in different age groups before, during, and after the COVID-19 pandemic, namely the transmission and death rates of influenza from the 40th week to the 17th week in 2016–2017, 2017–2018, and 2018–2019 (pre-pandemic), 2022–2023 (during the pandemic), and 2023–2024 (post-pandemic), as shown in Figs C–K of S1 Text and 1. Quantitative comparisons of average transmission and death rates across these periods are summarized in Table 1.

**Table 1:** The average transmission and death rates of each age group.

Index	Age \ Time	pre-pandemic			COVID-19 pandemic	post-pandemic
		2016-2017	2017-2018	2018-2019	2022-2023	2023-2024
Transmission rate	0-17	0.3570	0.2662	0.5981	0.3965	0.4793
	18-64	0.6504	0.5985	0.5070	0.3199	0.5588
	65+	3.5867	3.1906	1.2286	1.7995	2.9044
Death rate	0-17	0.0090	0.0099	0.0082	0.0138	0.0102
	18-64	0.0188	0.0244	0.0294	0.0305	0.0229
	65+	0.0464	0.0585	0.0544	0.0947	0.0622

We observe that the influenza transmission rate among the population aged 18 and above is relatively low in the last year of the COVID-19 pandemic (i.e., 2022–2023), with an average rate of 0.3199 for ages 18-64 and 1.7995 for ages 65+, compared to pre-pandemic averages of 0.6504 (2016–2017), 0.5985 (2017–2018), and 0.5070 (2018–2019) for ages 18–64, and 3.5867 (2016–2017), 3.1906 (2017–2018), and 1.2286 (2018–2019) for ages 65+. However, in the first year after the pandemic (i.e., 2023–2024), the influenza transmission rate among this age group has rebounded to pre-pandemic levels, reaching 0.5588 for ages 18–64 (95.4% recovery relative to pre-pandemic means) and 2.9044 for ages 65+ (surpassing the 2016–2019 baseline by 8.8%) (see Fig 2B and 2C and Table 1). The lower influenza transmission rates observed during the final years of the COVID-19 pandemic can be attributed to the implementation of NPIs and behavioral changes. The subsequent rebound to pre-pandemic levels in the first post-pandemic season (2023–2024) likely reflects the relaxation of NPIs, normalization of behaviors, and seasonal fluctuations in influenza transmission. Notably, the transmission rate among individuals under 18 years old remained stable across all periods, with pre-pandemic averages of 0.3570 (2016–2017), 0.2662 (2017–2018), and 0.5981 (2018–2019), compared to 0.4793 in 2023–2024 (post-pandemic), demonstrating no significant shift (see Fig 2A and Table 1).

Based on the estimated death rates, in the last year of the COVID-19 pandemic (i.e., 2022–2023), influenza mortality rates across all three age groups exhibited elevated levels: 0.0138 for ages 0–17 (53.3% increase over pre-pandemic means), 0.0305 for ages 18–64 (26% increase), and 0.0947 for ages 65+ (78.3% increase). However, in the first year after the pandemic (i.e., 2023-2024), influenza mortality rates in all three age groups reverted to pre-pandemic levels, declining to 0.0102 (−26.1% relative to 2022-2023) for ages 0-17, 0.0229 (−24.9%) for ages 18-64, and 0.0622 (−34.3%) for ages 65+ (Fig 2D, 2E, and 2F; Table 1). This phenomenon can be attributed to the disruption of global healthcare systems caused by the COVID-19 pandemic, which led to changes in healthcare delivery and prioritization. This disruption likely impacted the diagnosis, treatment, and prevention of other respiratory illnesses, including influenza. Consequently, factors such as delayed care, overwhelmed medical facilities, and altered healthcare-seeking behaviors may have contributed to the transient rise in influenza mortality rates during the pandemic period.

### Infectivity of influenza A and B

In this subsection, we use Model (3) to estimate the transmission rates of influenza A and B for specific time periods: from Week 40 of 2016 to Week 17 of 2017, from Week 40 of 2017 to Week 17 of 2018, from Week 40 of 2018 to Week 17 of 2019, from Week 40 of 2022 to Week 17 of 2023, and from Week 40 of 2023 to Week 17 of 2024 (see Figs L-T in S1 Text and 3). Quantitative comparisons of average transmission rates across these periods are summarized in Table 2.

**Table 2:** The average transmission rates of different influenza strains.

Index	Strain \ Time	pre-pandemic			COVID-19 pandemic	post-pandemic
		2016-2017	2017-2018	2018-2019	2022-2023	2023-2024
Transmission rate	Influenza A	1.8249	1.7713	1.9055	1.4525	1.9004
	Influenza B	1.9199	1.8937	1.8153	1.7935	1.8859

In terms of influenza A, a notable trend can be observed in its transmission rate. Prior to the COVID-19 pandemic, the average transmission rates during 2016–2017, 2017–2018, and 2018–2019 were 1.8249, 1.7713, and 1.9055, respectively. In the last year of the COVID-19 pandemic (2022–2023), the average transmission rate decreased to 1.4525, showing a reduction of approximately 26% compared to the pre-pandemic average (see Fig 4A). This decrease can be attributed to various factors, including improved hygiene practices, increased vaccination rates, and potential cross-reactive immunity from previous exposures to other respiratory viruses or the COVID-19 vaccine itself. In the first year after the COVID-19 pandemic (2023–2024), the average transmission rate rebounded to 1.9004, which is close to the pre-pandemic levels (see Fig 4A).

On the other hand, influenza B exhibits a slightly different pattern. The pre-pandemic average transmission rates in 2016–2017, 2017–2018, and 2018–2019 were 1.9199, 1.8937, and 1.8153, respectively. During the last year of the COVID-19 pandemic (2022–2023), the average transmission rate was 1.7935, and in the first year after the COVID-19 pandemic (2023–2024), it was 1.8859 (see Fig 4B). Compared to influenza A, the change in the average transmission rate of influenza B during the pandemic was relatively smaller. The difference between the pre-pandemic average (average of 2016–2019: 1.8763) and the average during the pandemic (average of 2022–2023: 1.7935) was only about 5% (see Fig 4B). This suggests that the factors influencing the transmission of influenza B may differ from those impacting influenza A, or that influenza B may be less sensitive to changes in public health measures or societal behaviors that have been implemented in response to the COVID-19 pandemic. Particularly, the B/Yamagata lineage has virtually disappeared after the pandemic, which may further indicate the distinct epidemiological dynamics of influenza B compared to influenza A.

### Effective reproduction numbers

To calculate the effective reproduction numbers of the two types of models, we substitute the estimated parameters into Eqs (2) and (4), as shown in Fig 5. We present the average values of the effective reproduction numbers in different time-periods in Table 3.

**Table 3:** The average value of effective reproduction numbers.

Reproduction Number \ Time	pre-pandemic			COVID-19 pandemic	post-pandemic
	2016-2017	2017-2018	2018-2019	2022-2023	2023-2024
${ℛ}_{e}^{AS}(t)$	1.1101	1.0900	1.1224	0.9745	1.0613
${ℛ}_{e}^{MS}(t)$	1.2431	1.1341	1.2000	1.2149	1.1703

Fig 5A shows the effective reproduction numbers (${ℛ}_{e}^{AS}(t)$) for the age-structured model, reflecting the transmission potential of influenza in different years. During the pre-pandemic periods (2016–2017, 2017–2018, and 2018–2019), the average values of the effective reproduction numbers were 1.1101, 1.0900, and 1.1224, respectively. The average pre-pandemic value of ${ℛ}_{e}^{AS}(t)$ is 1.1075. This relatively stable pre-pandemic average indicates a consistent transmission pattern. In the last year of the COVID-19 pandemic (2022-2023), the average value of ${ℛ}_{e}^{AS}(t)$ decreased to 0.9745, showing a decline of approximately 12% compared to the pre-pandemic average. This significant decrease potentially suggests alterations in influenza transmission dynamics due to factors such as changes in population behavior, immune status, or viral characteristics. In the first year after the pandemic (2023-2024), the average value of ${ℛ}_{e}^{AS}(t)$ was 1.0613, which is approaching the pre-pandemic level, with a difference of about 4.2% (see Table 3).

In contrast, Fig 5B shows the effective reproduction numbers (${ℛ}_{e}^{MS}(t)$) for the multi-strain model. The pre-pandemic average values of ${ℛ}_{e}^{MS}(t)$ in 2016–2017, 2017–2018, and 2018–2019 were 1.2431, 1.1341, and 1.2000, respectively. The average pre-pandemic value of ${ℛ}_{e}^{MS}(t)$ is 1.1924. During the last year of the COVID-19 pandemic (2022–2023), the average value of ${ℛ}_{e}^{MS}(t)$ was 1.2149, which is slightly higher than the pre-pandemic average by approximately 1.9%. In the first year after the pandemic (2023–2024), the average value of ${ℛ}_{e}^{MS}(t)$ was 1.1703, showing a decrease of about 1.9% compared to the pre-pandemic average (see Table 3). These differences in the trends and magnitudes of changes between ${ℛ}_{e}^{AS}(t)$ and ${ℛ}_{e}^{MS}(t)$ may reflect the interplay between strain-specific transmission characteristics and the broader impact of the COVID-19 pandemic on influenza dynamics.

## Discussion and conclusion

The COVID-19 pandemic has ushered in unprecedented transformations in societal structures and human behavior, significantly altering the community transmission patterns of respiratory viruses beyond SARS-CoV-2 [33]. However, the question remains as to whether the transmission patterns of non-SARS-CoV-2 respiratory infectious diseases, notably influenza, have undergone notable changes in the post-COVID-19 pandemic era. Data reported by the CDC [25,27,28] indicate that the peak of influenza infections and related deaths towards the end of 2022 surpassed pre-pandemic levels (see Figs A and B in S1 Text). To address this, we developed two types of infectious disease models: an age-structured model and a multi-strain model, both incorporating time-varying transmission and mortality rates [2,30].

Regarding the transmission and death rates of influenza in various age groups, we observed that the influenza transmission rate among adults aged 18 and above was relatively low in the last year of the COVID-19 pandemic but rebounded to pre-pandemic levels in the first year after the COVID-19 pandemic [10,34]. This rebound aligns with documented patterns of NPIs relaxation and behavioral normalization post-pandemic [3,6,7,35,36]. For instance, studies have shown that reduced social distancing and mask usage coincided with resurgent respiratory virus transmission [37]. In contrast, the transmission rate among individuals under 18 years old remained largely unchanged, consistent with reports of sustained in-person schooling and stable contact patterns among children during this period [38].

The elevated mortality rates during the final year of the COVID-19 pandemic may reflect systemic healthcare disruptions. Global reports indicate that the pandemic strained healthcare resources, causing delays in non-COVID care and significantly reducing influenza surveillance capacity [39–42]. For instance, surveillance data indicated that influenza testing declined by over 40% during the peak of the pandemic [43], potentially compromising timely diagnosis and treatment. The mortality decline to pre-pandemic levels observed in the first year of the COVID-19 pandemic aligns with the restoration of healthcare services and the reinstatement of public health priorities for influenza management [44].

In terms of the transmission rates of influenza A and B, we observed that influenza A exhibited a significant decline in transmission rate corresponding to the peak of new cases in the last year of the COVID-19 pandemic. Although it rebounded in the following year, which was the first year of the COVID-19 pandemic, it remained lower than the pre-pandemic levels. This decrease might be attributed to enhanced hygiene practices, elevated vaccination rates, and potential cross-reactive immunity. On the other hand, influenza B did not demonstrate a continuous downward trend in the first year after the COVID-19 pandemic and fluctuated between low and high values. The relative stability of influenza B transmission could reflect its shorter incubation period and different antigenic evolution patterns [45], though the near-extinction of the B/Yamagata lineage post-pandemic suggests additional strain-specific factors may be at play [10]. The differential strain responses underscore the need for subtype-specific modeling in pandemic preparedness strategies [46,47].

We also discovered that the effective reproduction numbers for both the age-structured and multi-strain models had relatively stable patterns in the pre-pandemic era. During the last year of the COVID-19 pandemic, significant deviations were observed. The changes in population behavior, such as reduced social mixing, along with modifications in immune status due to potential subclinical exposures to COVID-19 or other viruses, and possible alterations in the viral characteristics of influenza itself, all contributed to this shift. In the first year after the COVID-19 pandemic, the trend of effective reproduction numbers was gradually approaching the pre-pandemic pattern as life returned to normalcy, with people resuming their pre-pandemic social and travel habits [10,48].

In conclusion, the comparison of the infectivity, fatality, and effective reproduction numbers provides profound insights into the evolution of influenza transmission dynamics before and after the COVID-19 pandemic. The age-structured model emphasizes the crucial role of considering age as a determinant in understanding influenza transmission, while the multi-strain model highlights the added complexity introduced by the coexistence of multiple influenza strains. Our study elucidates the intricate interplay between diverse factors that shape influenza transmission and mortality, encompassing the COVID-19 pandemic, public health interventions, societal behaviors, and the unique traits of different influenza strains. These findings have the potential to guide the formulation of more effective public health strategies to mitigate the impact of influenza in the future.

Our study does possess several limitations. First, influenza surveillance data between weeks 18–39 annually were unavailable, along with strain-specific mortality data. Second, parameter values and initial conditions in our simulations represent informed estimates rather than precisely calibrated values. While these approximations may lack complete biological precision, they do not fundamentally affect the simulation outcomes. Third, while transmission dynamics are known to vary with geographic factors, population mobility patterns, environmental conditions, and climate change impacts, our current model structure and data availability constrained our ability to incorporate these multidimensional influences.

## Supporting information

S1 Text The details on the reproduction number and simulation results. (PDF)
