# Supplementary material for: Infectivity and fatality of influenza in pre- and post-COVID-19 pandemic year
Source: PLoS Comput Biol. 2025 Jul 8;21(7):e1013229. doi: 10.1371/journal.pcbi.1013229 (PMC12251249; doi:10.1371/journal.pcbi.1013229)
Supplement: S1 Text — (PDF) [file pcbi.1013229.s001.pdf]

Supporting Information (SI): Infectivity and fatality of influenza in pre- and post-COVID-19 pandemic year

Shuanglin Jing[1,2], Hao Wang[3,*]
[1]Department of Mathematics, Lanzhou Jiaotong University, Lanzhou, Gansu, 730070, China
[2]Research Center for Fundamental Disciplines of Complex System Analysis and Control, Lanzhou Jiaotong University, Lanzhou, Gansu, 730070, China
[3]Department of Mathematical and Statistical Sciences, University of Alberta, Edmonton, Alberta T6G 2R3, Canada

1 Reproduction number and parameter values of the model

Introducing the basic reproduction number $\mathcal{R}_0^{AS}$ for Model (1), we utilize the next generation matrix approach presented by van den Driessche and Watmough [1], assuming that both the transmission rate and the probability of deaths remain constant. Model (1) has a disease-free equilibrium

$$\mathcal{P}_0 = \left(S_1^0, S_2^0, \cdots, S_n^0, E_1^0, E_2^0, \cdots, E_n^0, I_1^0, I_2^0, \cdots, I_n^0, R_1^0, R_2^0, \cdots, R_n^0\right),$$

where

$$s_1^0 = \frac{\Lambda}{d_1 + \alpha_1},$$
$$s_k^0 = \frac{\Lambda \prod_{j=1}^{k-1} \alpha_j}{\prod_{j=1}^{k} (d_j + \alpha_j)},\ 2 \le k \le n,$$
$$e_k^0 = i_k^0 = r_k^0 = 0,\ 1 \le k \le n.$$

Taking the infected compartments e_k and i_k for $1 \le k \le n$, we obtain the Jacobian matrix of the infected class at the disease-free equilibrium as follows

$$\mathcal{F}_{AS} = \begin{bmatrix}
0 & 0 & 0 & \cdots & 0 & \beta_{11} & \beta_{12} & \beta_{13} & \cdots & \beta_{1n} \\
0 & 0 & 0 & \cdots & 0 & \beta_{21} & \beta_{22} & \beta_{23} & \cdots & \beta_{2n} \\
0 & 0 & 0 & \cdots & 0 & \beta_{31} & \beta_{32} & \beta_{33} & \cdots & \beta_{3n} \\
\vdots & \vdots & \vdots & \ddots & \vdots & \vdots & \vdots & \vdots & \ddots & \vdots \\
0 & 0 & 0 & \cdots & 0 & \beta_{n1} & \beta_{n2} & \beta_{n3} & \cdots & \beta_{nn} \\
0 & 0 & 0 & \cdots & 0 & 0 & 0 & 0 & \cdots & 0 \\
0 & 0 & 0 & \cdots & 0 & 0 & 0 & 0 & \cdots & 0 \\
0 & 0 & 0 & \cdots & 0 & 0 & 0 & 0 & \cdots & 0 \\
\vdots & \vdots & \vdots & \ddots & \vdots & \vdots & \vdots & \vdots & \ddots & \vdots \\
0 & 0 & 0 & \cdots & 0 & 0 & 0 & 0 & \cdots & 0
\end{bmatrix}$$

*Corresponding author. Email: hao8@ualberta.ca

and

$$\mathcal{V}_{AS} = \begin{bmatrix} V_{11}^{AS} & \mathbf{0} \\ V_{21}^{AS} & V_{22}^{AS} \end{bmatrix}$$

with

$$V_{11}^{AS} = \begin{bmatrix} \sigma_1 + d_1 + \alpha_1 & 0 & 0 & \cdots & 0 \\ -\alpha_1 & \sigma_2 + d_2 + \alpha_2 & 0 & \cdots & 0 \\ 0 & -\alpha_2 & \sigma_3 + d_3 + \alpha_3 & \cdots & 0 \\ \vdots & \vdots & \vdots & \ddots & \vdots \\ 0 & 0 & 0 & \cdots & \sigma_n + d_n + \alpha_n \end{bmatrix},$$

$$V_{21}^{AS} = \begin{bmatrix} -\sigma_1 & 0 & 0 & \cdots & 0 \\ 0 & -\sigma_2 & 0 & \cdots & 0 \\ 0 & 0 & -\sigma_3 & \cdots & 0 \\ \vdots & \vdots & \vdots & \ddots & \vdots \\ 0 & 0 & 0 & \cdots & -\sigma_n \end{bmatrix},$$

$$V_{22}^{AS} = \begin{bmatrix} \gamma_1 + d_1 + \alpha_1 & 0 & 0 & \cdots & 0 \\ -\alpha_1 & \gamma_2 + d_2 + \alpha_2 & 0 & \cdots & 0 \\ 0 & -\alpha_2 & \gamma_3 + d_3 + \alpha_3 & \cdots & 0 \\ \vdots & \vdots & \vdots & \ddots & \vdots \\ 0 & 0 & 0 & \cdots & \gamma_n + d_n + \alpha_n \end{bmatrix}.$$

According to the next generation matrix approach presented by van den Driessche and Watmough [1], we let ρ denote the spectral radius, the basic reproduction number, $\mathcal{R}_0^{AS}$, can be expressed as

$$\mathcal{R}_0^{AS} = \rho\big(\mathcal{F}\mathcal{V}^{-1}\big).$$

In epidemiology, the basic reproduction number is commonly used to measure the transmission potential at the beginning of an epidemic [2]. However, the transmission rate varies with time and is measured by the effective reproduction number. Therefore, the effective reproduction number is defined as

$$\mathcal{R}_e^{AS}(t) = \rho\big(\mathcal{F}_{AS}(t)\mathcal{V}_{AS}^{-1}\big), \tag{1}$$

where

$$\mathcal{F}_{AS}(t) = \begin{bmatrix} 0 & 0 & 0 & \cdots & 0 & \beta_{11}(t)\frac{S_1(t)}{N_1(t)} & \beta_{12}(t)\frac{S_1(t)}{N_2(t)} & \beta_{13}(t)\frac{S_1(t)}{N_3(t)} & \cdots & \beta_{1n}(t)\frac{S_1(t)}{N_n(t)} \\ 0 & 0 & 0 & \cdots & 0 & \beta_{21}(t)\frac{S_2(t)}{N_1(t)} & \beta_{22}(t)\frac{S_2(t)}{N_2(t)} & \beta_{23}(t)\frac{S_2(t)}{N_3(t)} & \cdots & \beta_{2n}(t)\frac{S_2(t)}{N_n(t)} \\ 0 & 0 & 0 & \cdots & 0 & \beta_{31}(t)\frac{S_3(t)}{N_1(t)} & \beta_{32}(t)\frac{S_3(t)}{N_2(t)} & \beta_{33}(t)\frac{S_3(t)}{N_3(t)} & \cdots & \beta_{3n}(t)\frac{S_3(t)}{N_n(t)} \\ \vdots & \vdots & \vdots & \ddots & \vdots & \vdots & \vdots & \vdots & \ddots & \vdots \\ 0 & 0 & 0 & \cdots & 0 & \beta_{n1}(t)\frac{S_n(t)}{N_1(t)} & \beta_{n2}(t)\frac{S_n(t)}{N_2(t)} & \beta_{n3}(t)\frac{S_n(t)}{N_3(t)} & \cdots & \beta_{nn}(t)\frac{S_n(t)}{N_n(t)} \\ 0 & 0 & 0 & \cdots & 0 & 0 & 0 & 0 & \cdots & 0 \\ 0 & 0 & 0 & \cdots & 0 & 0 & 0 & 0 & \cdots & 0 \\ 0 & 0 & 0 & \cdots & 0 & 0 & 0 & 0 & \cdots & 0 \\ \vdots & \vdots & \vdots & \ddots & \vdots & \vdots & \vdots & \vdots & \ddots & \vdots \\ 0 & 0 & 0 & \cdots & 0 & 0 & 0 & 0 & \cdots & 0 \end{bmatrix}.$$

Similarly, according to the next generation matrix approach presented by van den Driessche and Watmough [1], the effective reproduction number of Model (3) is defined as

$$\mathcal{R}_e^{MS}(t) = \rho\big(\mathcal{F}_{MS}(t)\mathcal{V}_{MS}^{-1}\big), \tag{2}$$

where

$$\mathcal{F}_{MS}(t)=\begin{bmatrix} 0 & 0 & 0 & \cdots & 0 & \beta_1(t)\frac{S(t)}{N(t)} & 0 & 0 & \cdots & 0\\ 0 & 0 & 0 & \cdots & 0 & 0 & \beta_2(t)\frac{S(t)}{N(t)} & 0 & \cdots & 0\\ 0 & 0 & 0 & \cdots & 0 & 0 & 0 & \beta_3(t)\frac{S(t)}{N(t)} & \cdots & 0\\ \vdots & \vdots & \vdots & \ddots & \vdots & \vdots & \vdots & \vdots & \ddots & \vdots\\ 0 & 0 & 0 & \cdots & 0 & 0 & 0 & 0 & \cdots & \beta_n(t)\frac{S(t)}{N(t)}\\ 0 & 0 & 0 & \cdots & 0 & 0 & 0 & 0 & \cdots & 0\\ 0 & 0 & 0 & \cdots & 0 & 0 & 0 & 0 & \cdots & 0\\ 0 & 0 & 0 & \cdots & 0 & 0 & 0 & 0 & \cdots & 0\\ \vdots & \vdots & \vdots & \ddots & \vdots & \vdots & \vdots & \vdots & \ddots & \vdots\\ 0 & 0 & 0 & \cdots & 0 & 0 & 0 & 0 & \cdots & 0 \end{bmatrix}.$$

and

$$\mathcal{V}_{MS}=\begin{bmatrix} V_{11}^{MS} & \mathbf{0}\\ V_{21}^{MS} & V_{22}^{MS} \end{bmatrix}$$

with

$$V_{11}^{MS}=\begin{bmatrix} \sigma_1+d & 0 & 0 & \cdots & 0\\ 0 & \sigma_2+d & 0 & \cdots & 0\\ 0 & 0 & \sigma_3+d & \cdots & 0\\ \vdots & \vdots & \vdots & \ddots & \vdots\\ 0 & 0 & 0 & \cdots & \sigma_n+d \end{bmatrix}, V_{21}^{MS}=\begin{bmatrix} -\sigma_1 & 0 & 0 & \cdots & 0\\ 0 & -\sigma_2 & 0 & \cdots & 0\\ 0 & 0 & -\sigma_3 & \cdots & 0\\ \vdots & \vdots & \vdots & \ddots & \vdots\\ 0 & 0 & 0 & \cdots & -\sigma_n \end{bmatrix},$$

$$V_{22}^{MS}=\begin{bmatrix} \gamma_1+d & 0 & 0 & \cdots & 0\\ 0 & \gamma_2+d & 0 & \cdots & 0\\ 0 & 0 & \gamma_3+d & \cdots & 0\\ \vdots & \vdots & \vdots & \ddots & \vdots\\ 0 & 0 & 0 & \cdots & \gamma_n+d \end{bmatrix}.$$

Table A: The parameter description of Model (1).

Parameters	Description (Units)	Value	Source
Λ	The recruitment rate of susceptible individuals (number/week)	69230	[3]
$1/\sigma_k$	The latent period length of individuals in age-group k (week)	2/7	[4]
δ_k	The rate at which a recovered individual loses immunity (becoming susceptible again)(1/week)	1/52	[5]
α_k	The rate at which individuals of age-group k transfer into age-group $k+1$ (1/week)	$\alpha_1 = 1/(18 \times 52)$<br>$\alpha_2 = 1/(46 \times 52)$<br>$\alpha_3 = 0$	estimated
d_k	The natural mortality rate in age-group k (1/week)	$d_1 = 1/(76 \times 52)$<br>$d_2 = 1/(58 \times 52)$<br>$d_2 = 1/(12 \times 52)$	[3]
$1/\gamma_k$	The infectious period in age-group k (week)	1	[4]
$\mu_k(t)$	Probability of deaths among infected individuals (dimensionless)	Panels D-F of Figs G-K	estimated
$\beta_{kj}(t)$	Transmission rate between individuals in age-group k and individuals in age-group j (1/week)	Panels A-C of Figs G-K	MCMC
$S_k(0)$	The initial value of susceptible individuals in the k-th age group	$S_1(0) = 82600000 \times 0.55$<br>$S_2(0) = 197700000 \times 0.5$<br>$S_3(0) = 59800000 \times 0.3$	[3, 6]
$E_k(0)$	The initial value of latent individuals in the k-th age group	$E_k(0) = \frac{I_k(0)}{\sigma_k}$	estimated
$I_k(0)$	The initial value of infected individuals in the k-th age group	The number of new cases at the initial time	[7]
$R_k(0)$	The initial value of recovered individuals in the k-th age group	$R_1(0) = 82600000 \times 0.45$<br>$R_2(0) = 197700000 \times 0.5$<br>$R_3(0) = 59800000 \times 0.7$	[3, 6]

Table B: The parameter description of Model (3).

Parameters	Description (Units)	Value	Source
Λ	The recruitment rate of susceptible individuals (number/week)	69230	[3]
$1/\sigma_i$	The latent period length of individuals infected with strain i (week)	2/7	[4]
δ	The rate at which a recovered individual loses immunity (becoming susceptible again)(1/week)	1/52	[5]
d	The natural mortality rate (1/week)	$d = 1/(76 \times 52)$	[3]
$1/\gamma_i$	The infectious period length of individuals infected with strain i (week)	1	[4]
$\mu_i(t)$	Probability of deaths of individuals infected with strain i (dimensionless)	0	estimated
$\beta_i(t)$	Transmission rate of individuals infected with strain i (1/week)	Figs P-T	MCMC
$S(0)$	The initial value of susceptible individuals	$S(0) = 82600000 \times 0.55$ $+197700000 \times 0.5$ $+59800000 \times 0.3$	[3, 6]
$E_i(0)$	The initial value of latent individuals infected with strain i	$E_i(0) = \frac{I_i(0)}{\sigma_i}$	estimated
$I_i(0)$	The initial value of infected individuals infected with strain i	The number of new cases at the initial time	[8]
$R(0)$	The initial value of recovered individuals	$R(0) = 82600000 \times 0.45$ $+197700000 \times 0.5$ $+59800000 \times 0.7$	[3, 6]

2 Influenza data in the United States

The data presents the temporal trends of influenza cases and deaths across three age groups (0-17, 18-64, and 65+) (see Fig A) and the circulation dynamics of influenza strains (A subtypes H1N12009, H3, and unspecified; B lineages Yamagata, Victoria, and unspecified) in the United States from October 2016 to September 2024 (see Fig B). Trends are contextualized within pre-pandemic, pandemic, and post-pandemic COVID-19 phases [9, 10], with annual gaps in case reporting between Weeks 18-39 reflecting periods of historically low influenza activity. Mortality and case patterns are stratified by age, while strain-specific incidence highlights shifts in predominant subtypes and lineages over time.

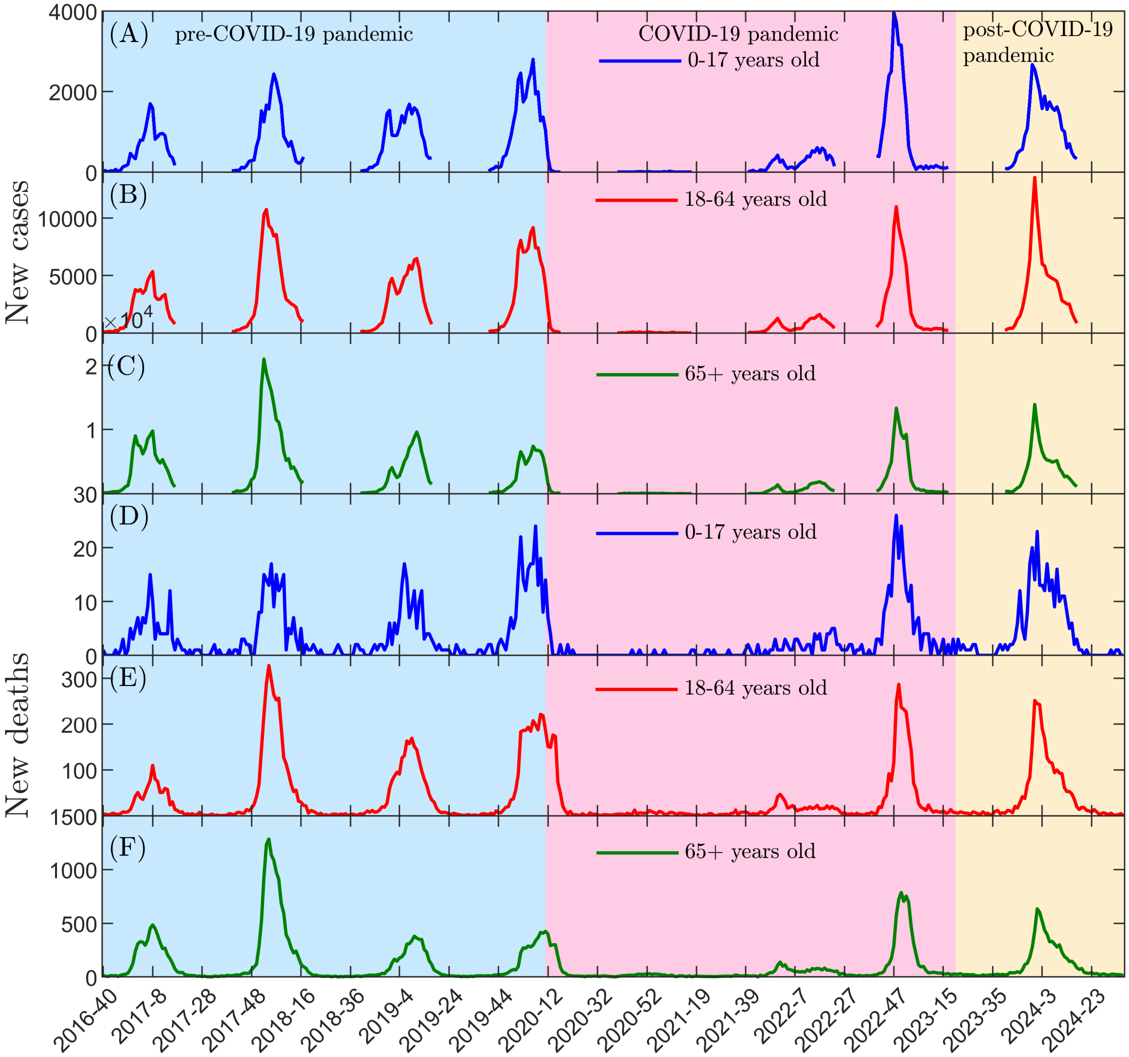

Fig A: The temporal variation of new flu cases and deaths among populations aged 0-17, 18-64, and over 65 in the United States from Week 40 of 2016 to Week 36 of 2024. Panels A, B, and C show the new cases of influenza among individuals aged 0-17, 18-64, and 65 +, respectively. Panels D, E, and F show the new deaths of influenza, in the same order. The light blue areas, pink areas, and yellow areas represent the pre-pandemic, pandemic, and post-pandemic periods of COVID-19 [9, 10], respectively. Note that the data on new cases from Week 18 to Week 39 of each year is missing, however, influenza is transmitted at a low level during this period.

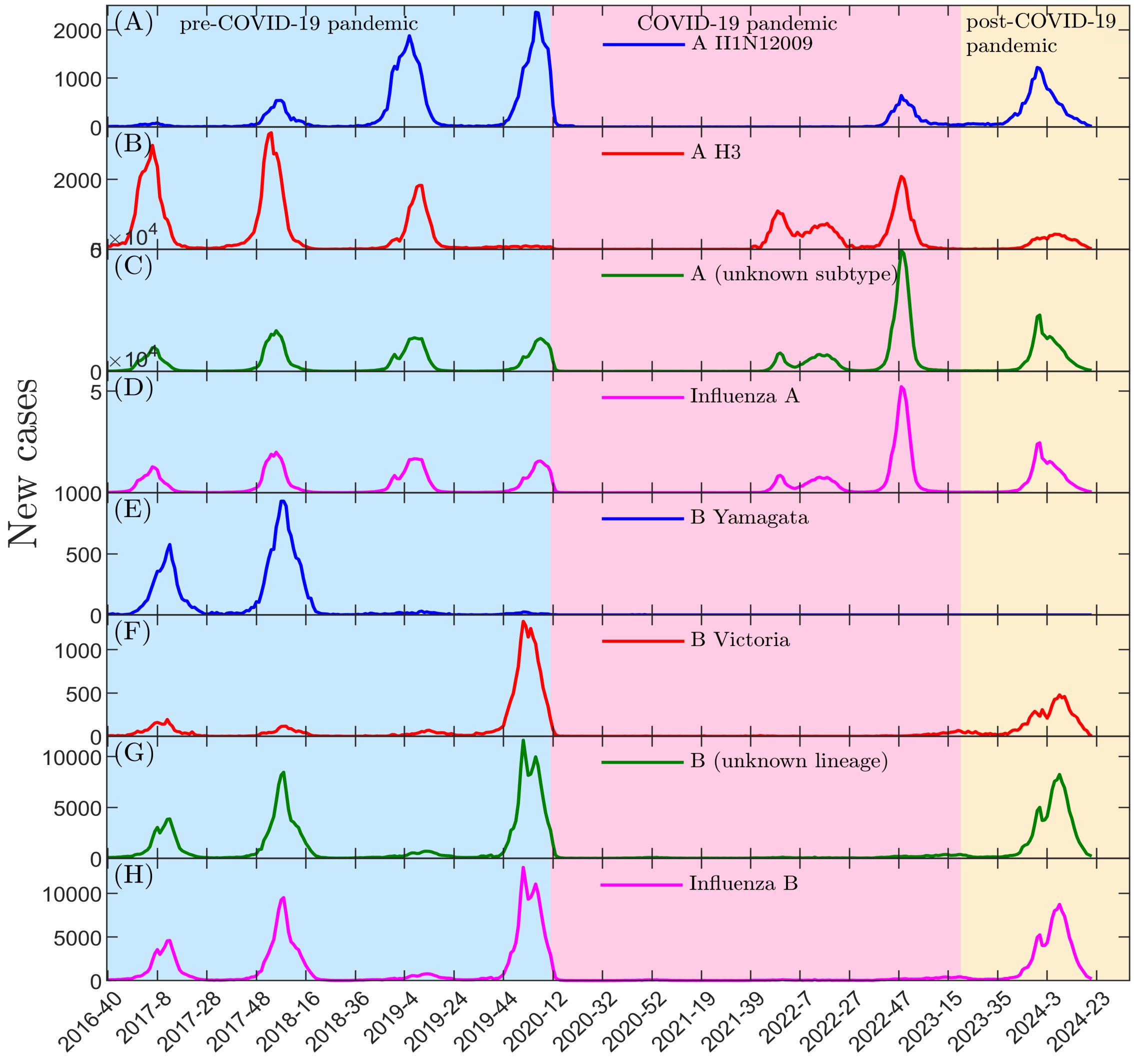

Fig B: The temporal variation of new cases of infection with different influenza strains in the United States from the 40th week of 2016 to the 36th week of 2024. Panels A, B, C, and D show new cases of infection with strain A H1N12009, strain A H3, strain A (unknown subtype) and all strains A, respectively. Panels E, F, G, and H show new cases of infection with strain B Yamagata, strain B Victoria, strain B (unknown lineage) and all strains B, respectively. The light blue areas, pink areas, and a yellow areas represent the pre-COVID-19 pandemic, COVID-19 pandemic, and post-COVID-19 pandemic [9, 10], respectively.

3 Simulation results of age-structure model

In this section, the age-structure model fitting based on data pertaining to influenza cases and deaths across three distinct age groups in the United States (0-17 years, 18-64 years, and over 65 years) for five different time periods: from the 40th week of 2016 to the 17th week of 2017 (see Fig C), the 40th week of 2017 to the 17th week of 2018 (see Fig D), the 40th week of 2018 to the 17th week of 2019 (see Fig E), the 40th week of 2022 to the 17th week of 2023 (see Fig F), and the 40th week of 2023 to the 17th week of 2024 (see Fig 1 in the main text). For each period, panels A, B, and C of Figs G-K exhibit the fitted transmission rates among influenza infected populations within the specified age brackets, utilizing weekly influenza hospitalization surveillance case data. Concurrently, panels D, E, and F of Figs G-K depict the fitted death rates for the same age groups, relying on weekly counts of new deaths. Notably, the transmission rates in these models are represented as cubic spline functions with three nodes ($n_\beta = 3$), providing a flexible and smooth estimation of the transmission dynamics across the observed time frames. In Figs C-F and 1, the estimated cases in the top three figures are obtained from the $C_k^{AS}(j)$ variable in the Model (1), and the estimated cases in the bottom three figures are obtained from the $U_k^{AS}(j)$ variable in the Model (1).

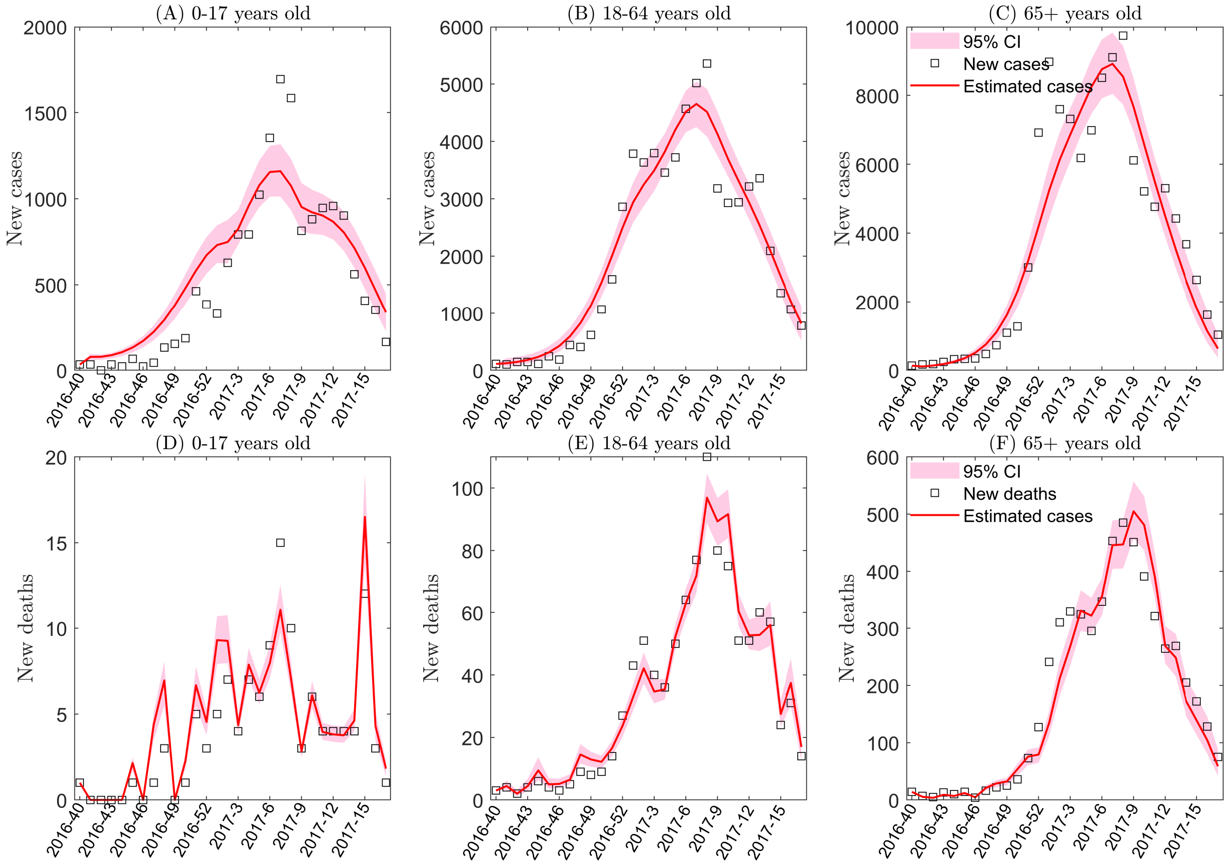

Fig C: Age-structure model fitting based on data for influenza case and deaths from the 40th week of 2016 to the 17th week of 2017 for three age groups in the United States (i.e. 0-17 years old, 18-64 years old, and over 65 years old). Panels A, B, and C show the transmission rates fitting of influenza infected populations aged 0-17, 18-64, and 65+, respectively, based on the weekly number of influenza hospitalization surveillance cases. Panels D, E, and F show the fitting of the death rates (in the same order), based on weekly numbers of new deaths.

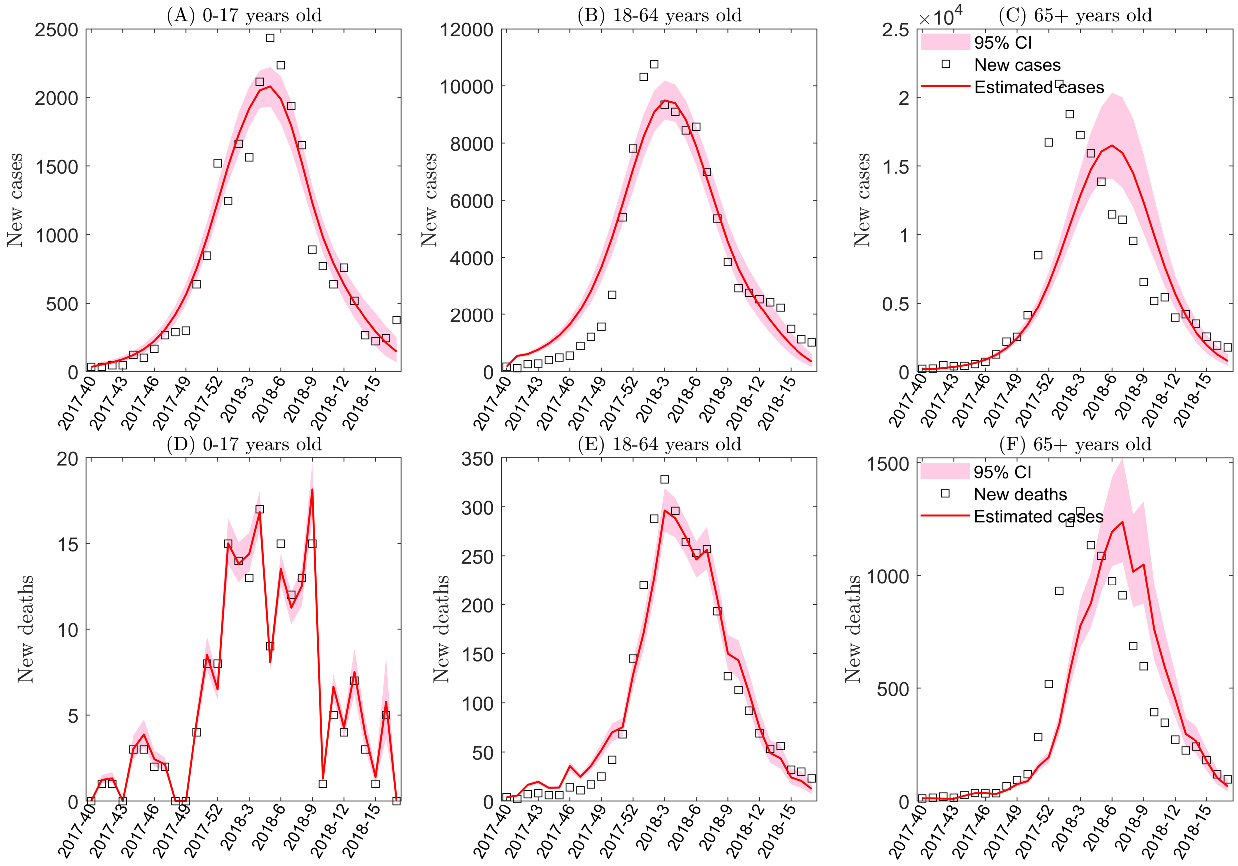

Fig D: Age-structure model fitting based on data for influenza case and deaths from the 40th week of 2017 to the 17th week of 2018 for three age groups in the United States (i.e. 0-17 years old, 18-64 years old, and over 65 years old). Panels A, B, and C show the transmission rates fitting of influenza infected populations aged 0-17, 18-64, and 65+, respectively, based on the weekly number of influenza hospitalization surveillance cases. Panels D, E, and F show the fitting of the death rates (in the same order), based on weekly numbers of new deaths.

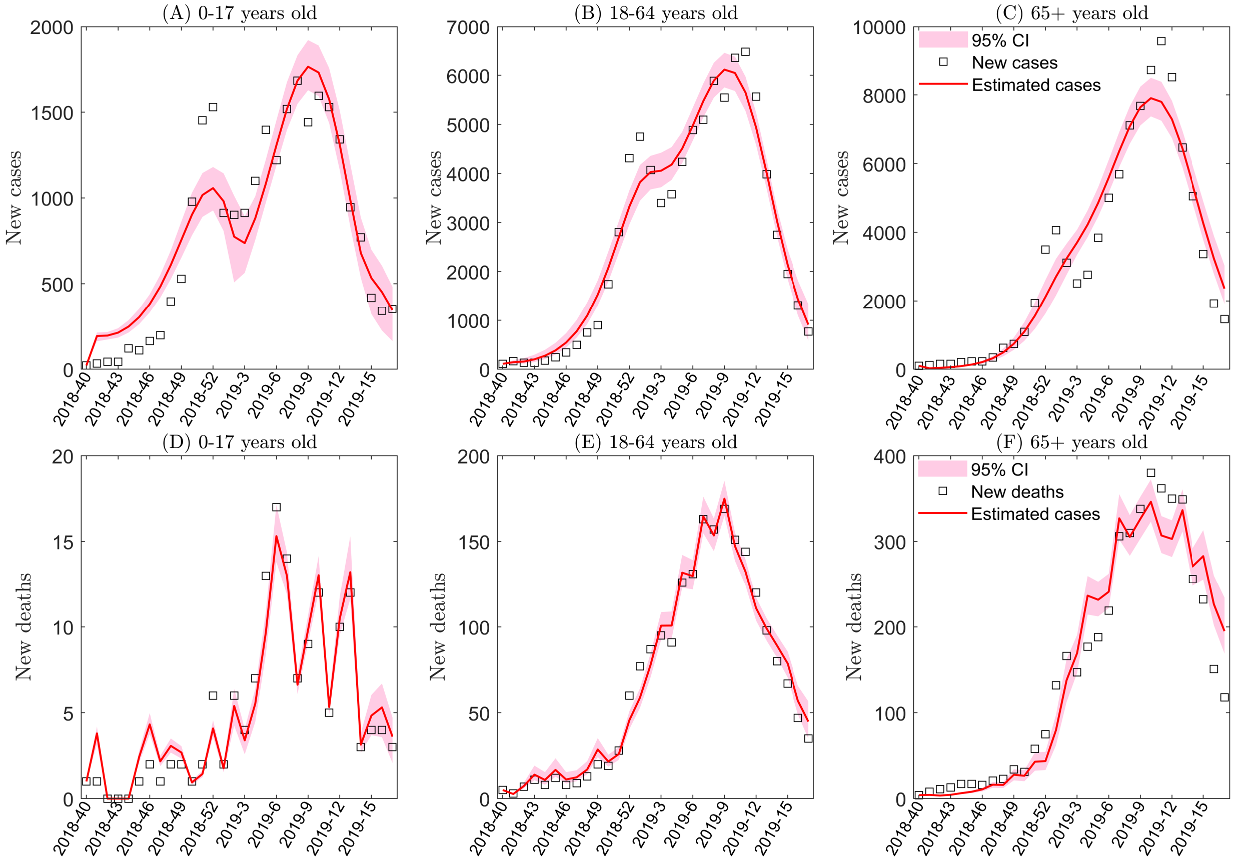

Fig E: Age-structure model fitting based on data for influenza case and deaths from the 40th week of 2018 to the 17th week of 2019 for three age groups in the United States (i.e. 0-17 years old, 18-64 years old, and over 65 years old). Panels A, B, and C show the transmission rates fitting of influenza infected populations aged 0-17, 18-64, and 65+, respectively, based on the weekly number of influenza hospitalization surveillance cases. Panels D, E, and F show the fitting of the death rates (in the same order), based on weekly numbers of new deaths.

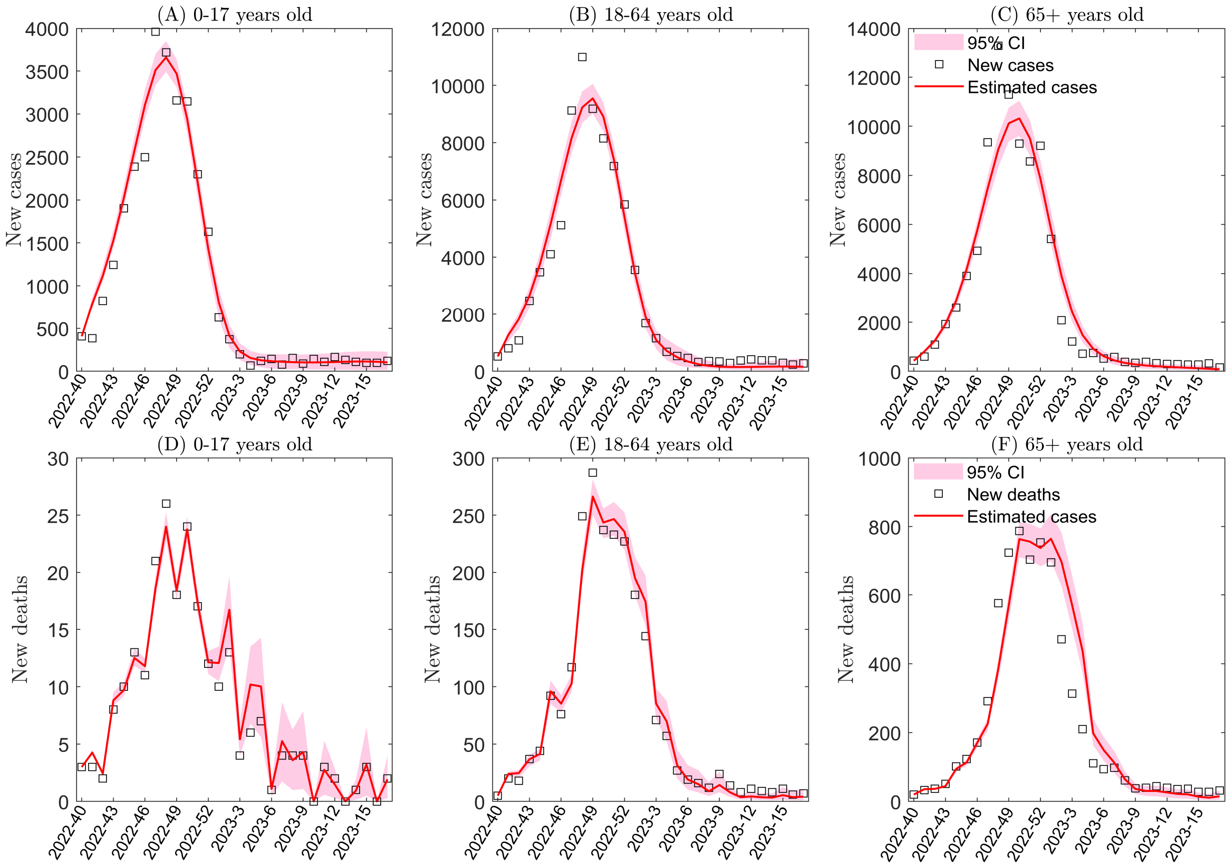

Fig F: Age-structure model fitting based on data for influenza case and deaths from the 40th week of 2022 to the 17th week of 2023 for three age groups in the United States (i.e. 0-17 years old, 18-64 years old, and over 65 years old). Panels A, B, and C show the transmission rates fitting of influenza infected populations aged 0-17, 18-64, and 65+, respectively, based on the weekly number of influenza hospitalization surveillance cases. Panels D, E, and F show the fitting of the death rates (in the same order), based on weekly numbers of new deaths.

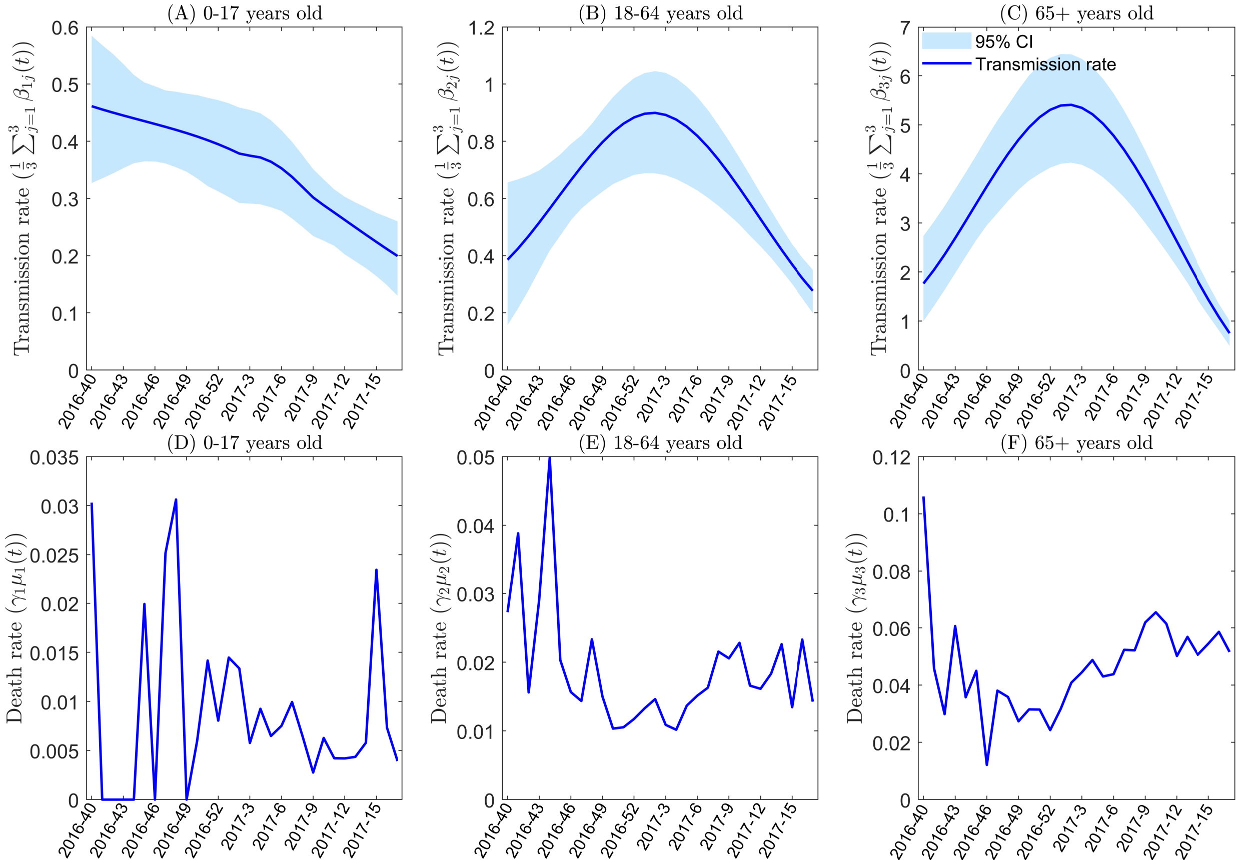

Fig G: Age-specific transmission and death rate profiles for influenza from the 40th week of 2016 to the 17th week of 2017 in the United States. Panels A-C present reconstructed transmission rates for (A) 0-17 year old, (B) 18-64 year old, and (C) adults 65+, derived from hospitalization surveillance data through cubic spline fitting ($n_\beta = 3$ nodes). Panels D-F show corresponding age-stratified death rates for (D) 0-17, (E) 18-64, and (F) 65+ populations, estimated from weekly death counts.

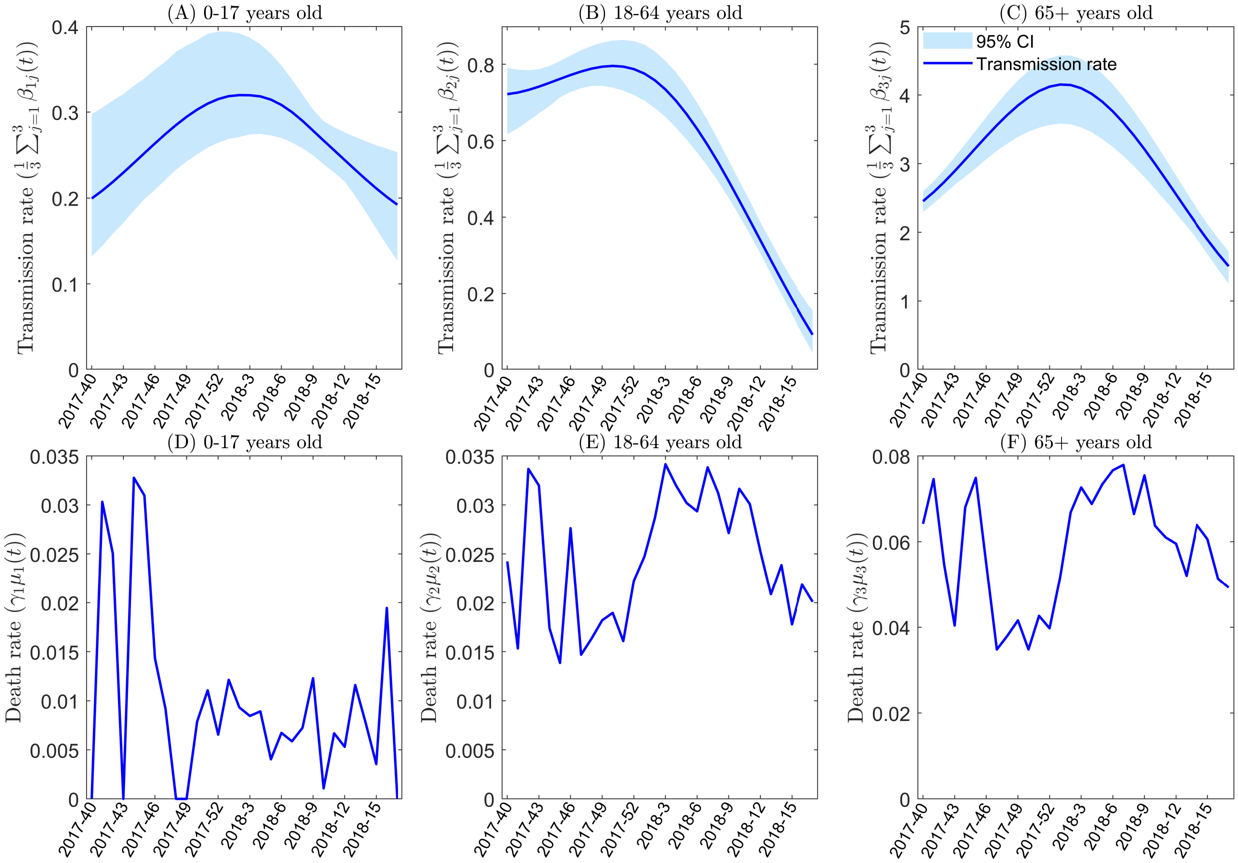

Fig H: Age-specific transmission and death rate profiles for influenza from the 40th week of 2017 to the 17th week of 2018 in the United States. Panels A-C present reconstructed transmission rates for (A) 0-17 year old, (B) 18-64 year old, and (C) adults 65+, derived from hospitalization surveillance data through cubic spline fitting ($n_\beta = 3$ nodes). Panels D-F show corresponding age-stratified death rates for (D) 0-17, (E) 18-64, and (F) 65+ populations, estimated from weekly death counts.

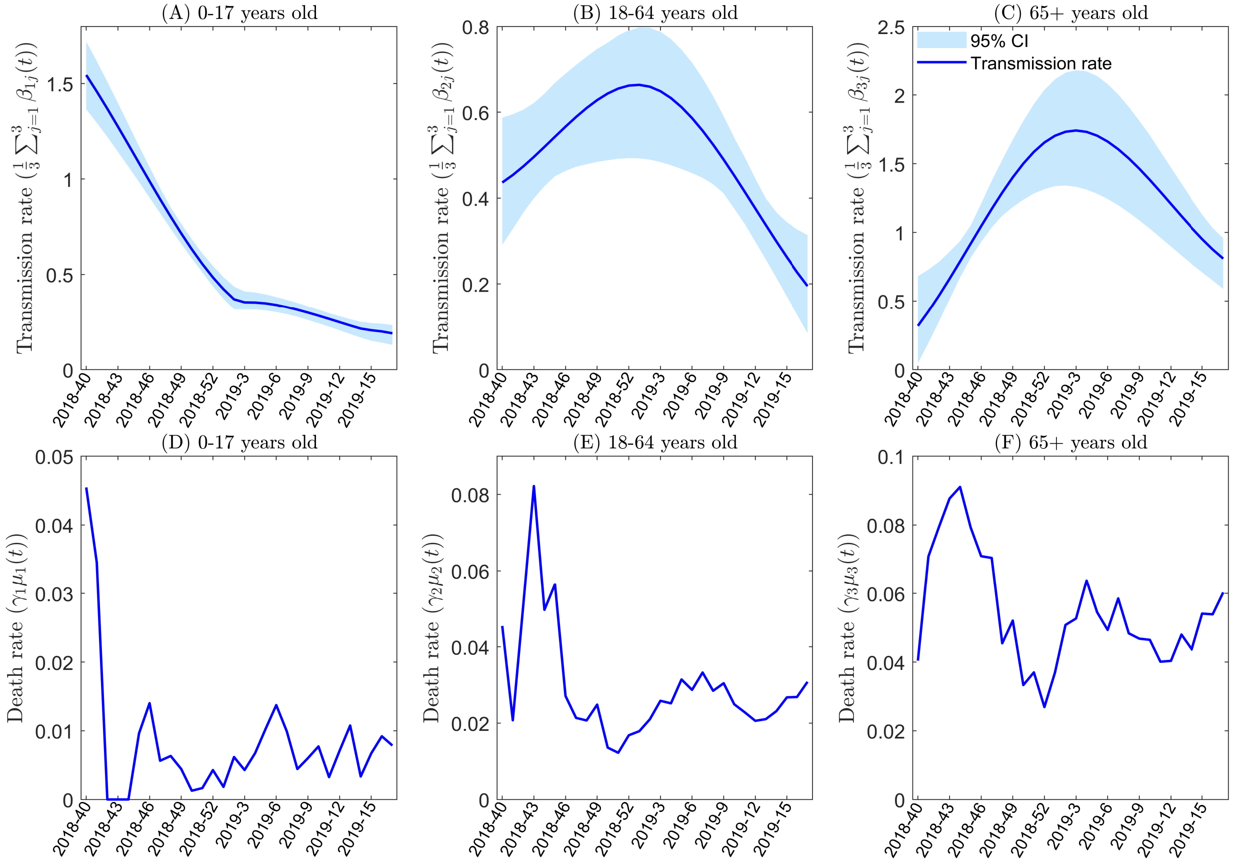

Fig I: Age-specific transmission and death rate profiles for influenza from the 40th week of 2018 to the 17th week of 2019 in the United States. Panels A-C present reconstructed transmission rates for (A) 0-17 year old, (B) 18-64 year old, and (C) adults 65+, derived from hospitalization surveillance data through cubic spline fitting ($n_\beta = 3$ nodes). Panels D-F show corresponding age-stratified death rates for (D) 0-17, (E) 18-64, and (F) 65+ populations, estimated from weekly death counts.

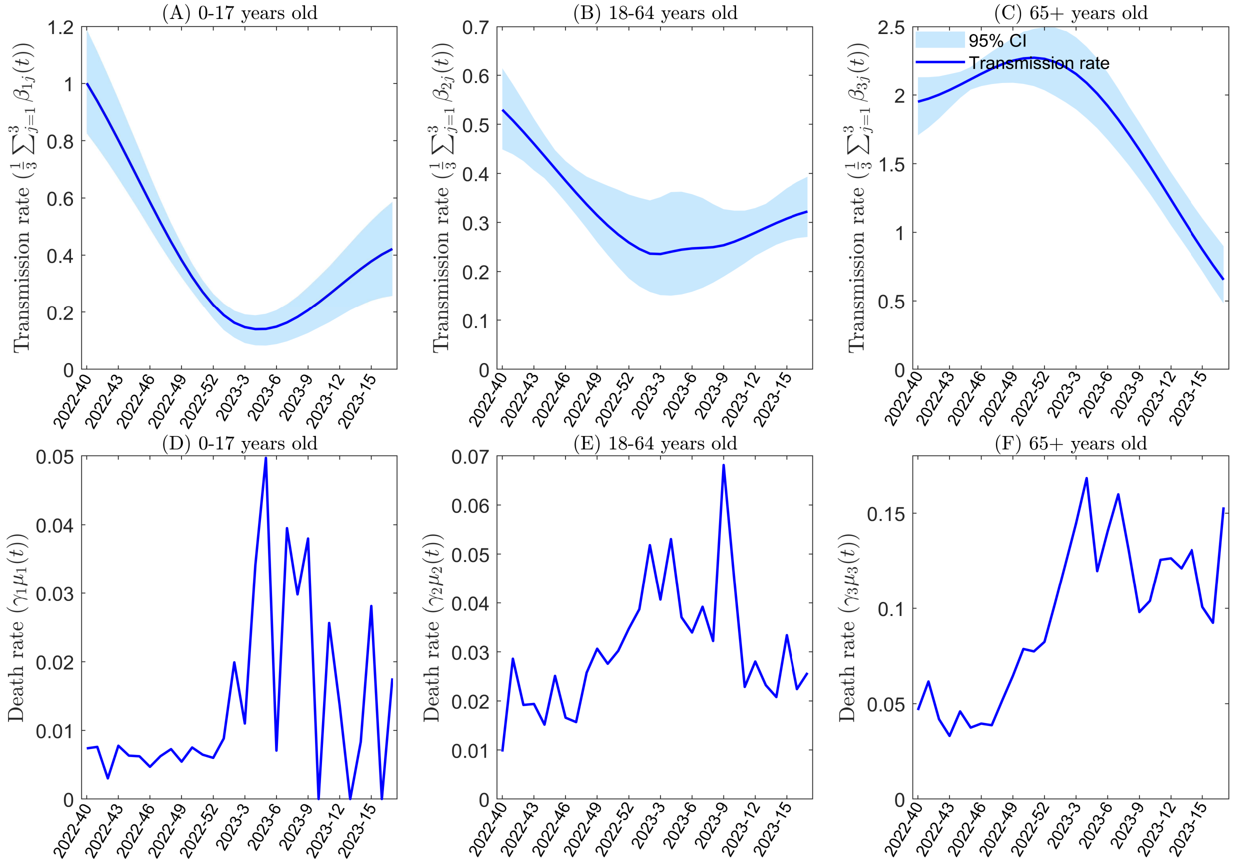

Fig J: Age-specific transmission and death rate profiles for influenza from the 40th week of 2022 to the 17th week of 2023 in the United States. Panels A-C present reconstructed transmission rates for (A) 0-17 year old, (B) 18-64 year old, and (C) adults 65+, derived from hospitalization surveillance data through cubic spline fitting ($n_\beta = 3$ nodes). Panels D-F show corresponding age-stratified death rates for (D) 0-17, (E) 18-64, and (F) 65+ populations, estimated from weekly death counts.

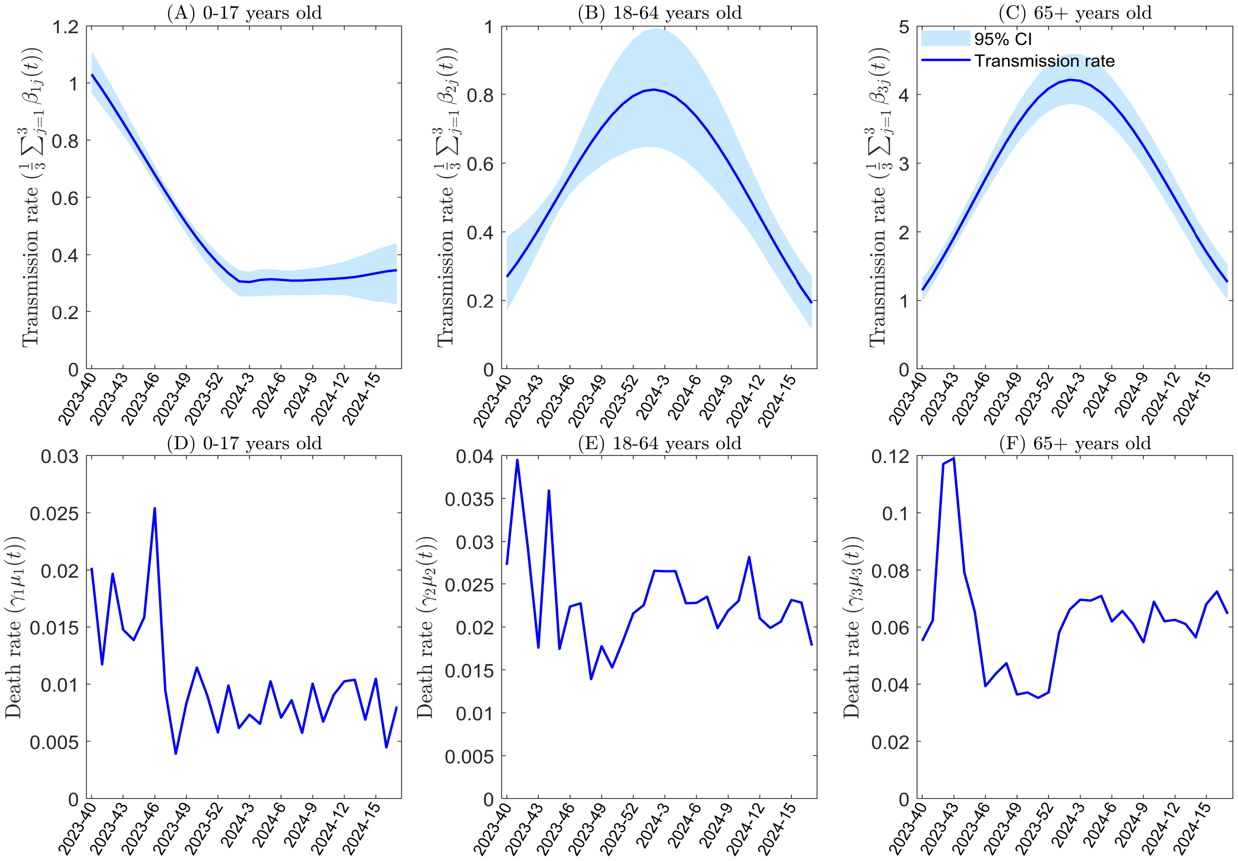

Fig K: Age-specific transmission and death rate profiles for influenza from the 40th week of 2023 to the 17th week of 2024 in the United States. Panels A-C present reconstructed transmission rates for (A) 0-17 year old, (B) 18-64 year old, and (C) adults 65+, derived from hospitalization surveillance data through cubic spline fitting ($n_\beta = 3$ nodes). Panels D-F show corresponding age-stratified death rates for (D) 0-17, (E) 18-64, and (F) 65+ populations, estimated from weekly death counts.

4 Simulation results of multi-strain model

In this section, we conduct the study which involves multi-strain model fitting based on data for influenza A and B cases in the United States from different time periods. Specifically, it includes data from the 40th week of 2016 to the 17th week of 2017 (see Fig L), the 40th week of 2017 to the 17th week of 2018 (see Fig M), the 40th week of 2018 to the 17th week of 2019 (see Fig N), the 40th week of 2022 to the 17th week of 2023 (see Fig O), and the 40th week of 2023 to the 17th week of 2024 (see Fig 2 in the main text). For each of these time ranges, panels A and B of Figs P-T show the transmission rates fitting of influenza A and B, respectively. The transmission rates are taken to be cubic spline functions, with the number of nodes n_β equal to three, and are based on the weekly number of influenza cases detected by sentinel and non-sentinel surveillance. In Figs L-O and 2, the estimated cases are obtained from the $C_i^{MS}(j)$ variable in the Model (3).

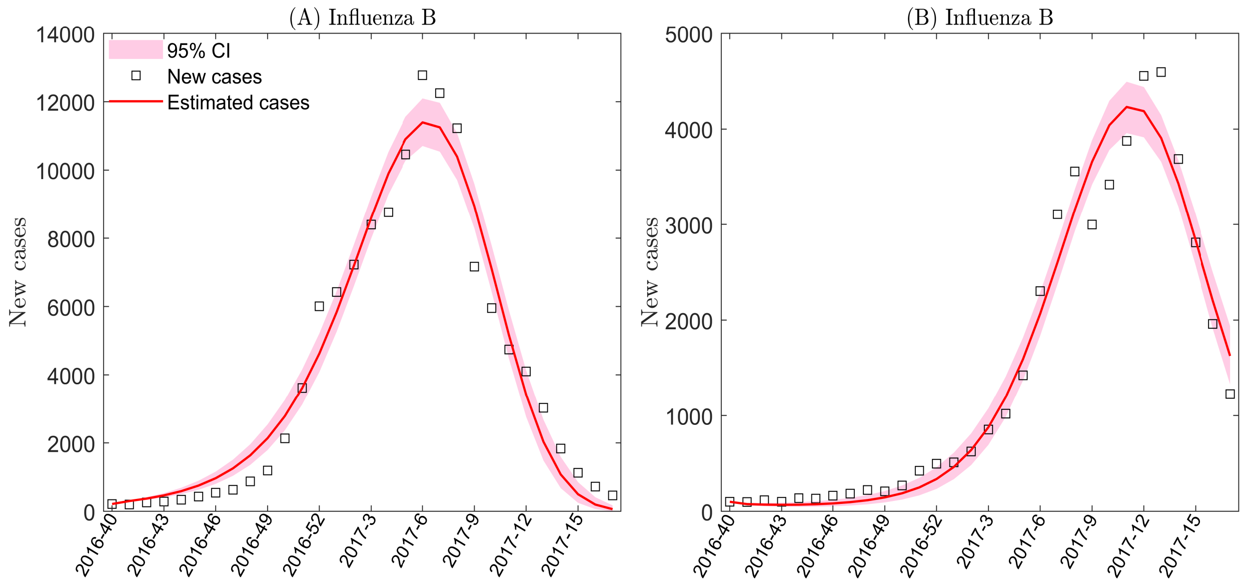

Fig L: Multi-strain model fitting based on data for influenza A and B cases from the 40th week of 2016 to the 17th week of 2017 in the United States. Panels A and B show the transmission rates fitting of influenza A and B, respectively, based on the weekly number of influenza cases detected by sentinel and non-sentinel surveillance.

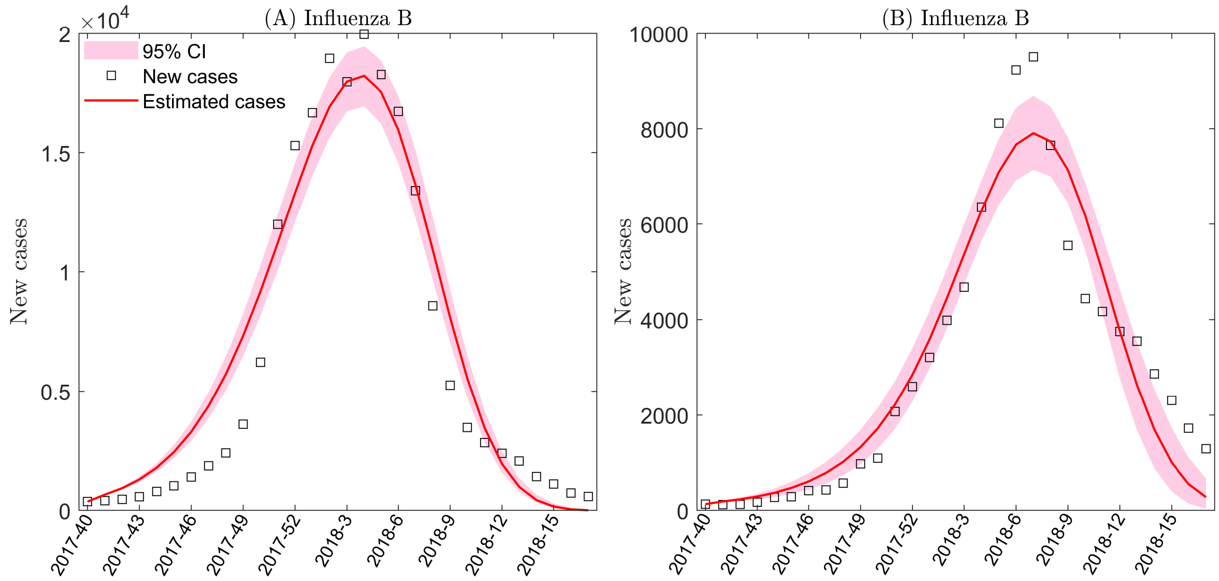

Fig M: Multi-strain model fitting based on data for influenza A and B cases from the 40th week of 2017 to the 17th week of 2018 in the United States. Panels A and B show the transmission rates fitting of influenza A and B, respectively, based on the weekly number of influenza cases detected by sentinel and non-sentinel surveillance.

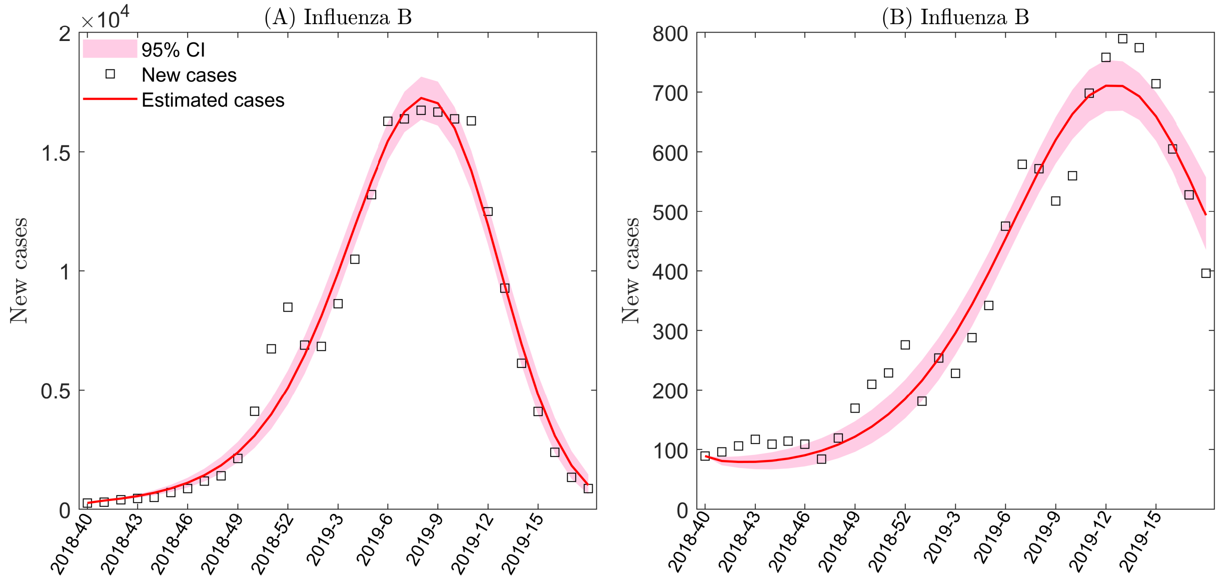

Fig N: Multi-strain model fitting based on data for influenza A and B cases from the 40th week of 2018 to the 17th week of 2019 in the United States. Panels A and B show the transmission rates fitting of influenza A and B, respectively, based on the weekly number of influenza cases detected by sentinel and non-sentinel surveillance.

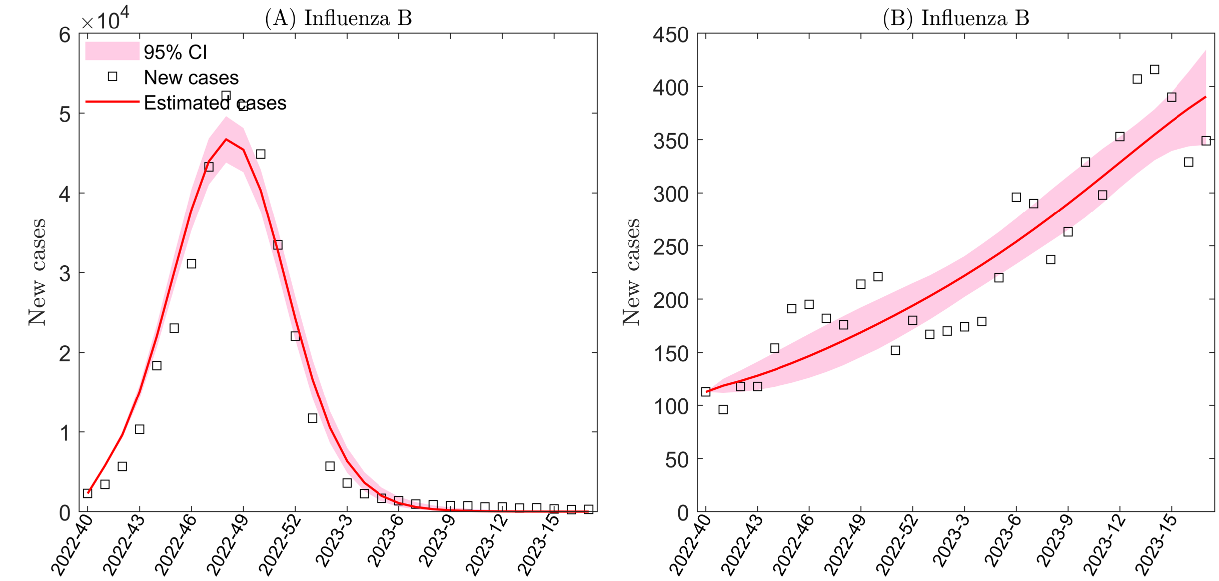

Fig O: Multi-strain model fitting based on data for influenza A and B cases from the 40th week of 2022 to the 17th week of 2023 in the United States. Panels A and B show the transmission rates fitting of influenza A and B, respectively, based on the weekly number of influenza cases detected by sentinel and non-sentinel surveillance.

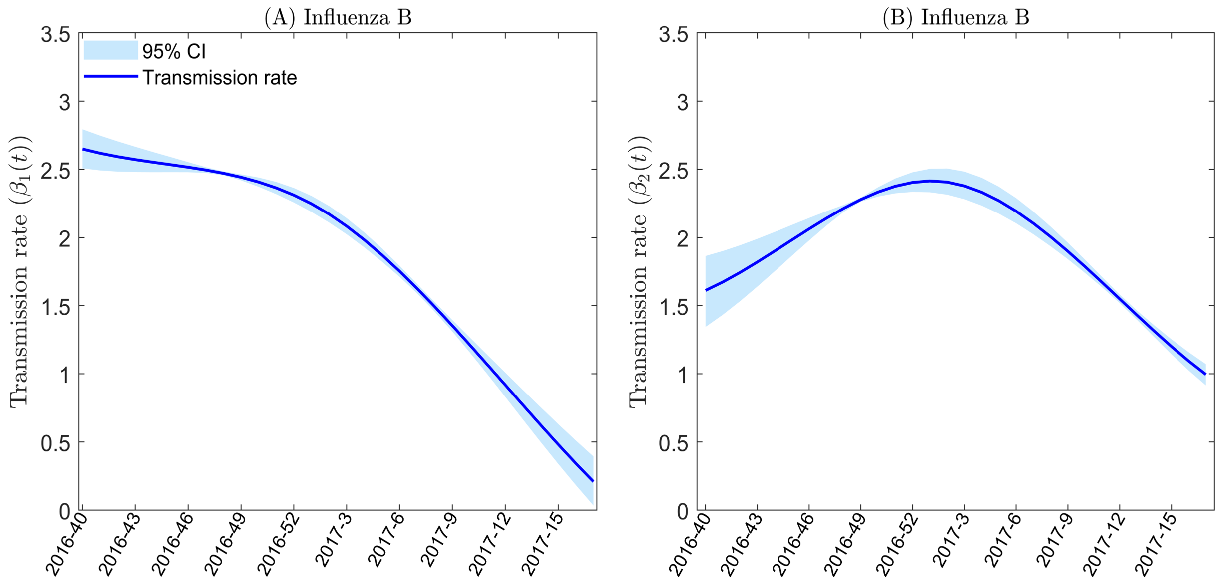

Fig P: Estimated time-varying transmission rates of influenza A and B in the United States (week 40 of 2016 to week 17 of 2017). Panels A and B show the modeled transmission rates for influenza A and B strains, respectively, derived from cubic spline functions ($n_\beta = 3$).

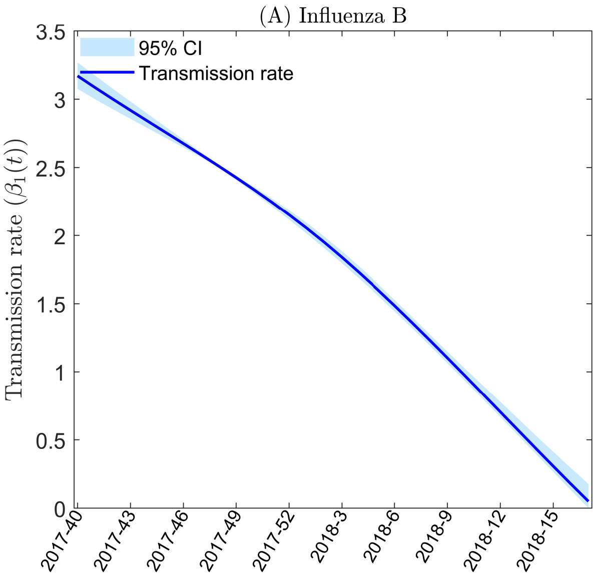

Fig Q: Estimated time-varying transmission rates of influenza A and B in the United States (week 40 of 2017 to week 17 of 2018). Panels A and B show the modeled transmission rates for influenza A and B strains, respectively, derived from cubic spline functions ($n_\beta = 3$).

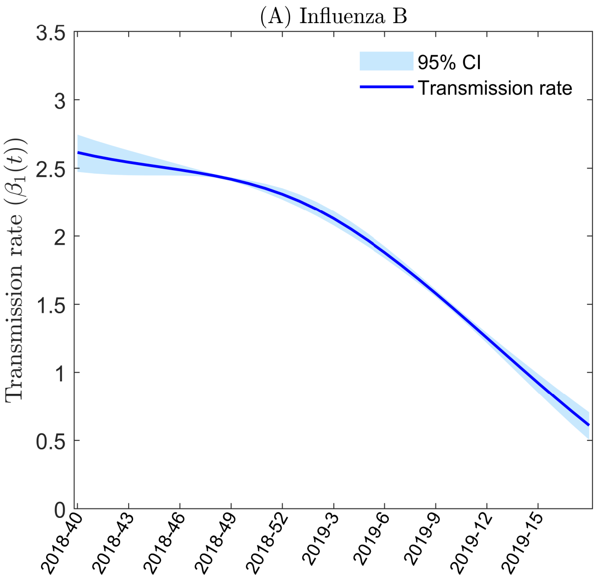

Fig R: Estimated time-varying transmission rates of influenza A and B in the United States (week 40 of 2018 to week 17 of 2019). Panels A and B show the modeled transmission rates for influenza A and B strains, respectively, derived from cubic spline functions ($n_\beta = 3$).

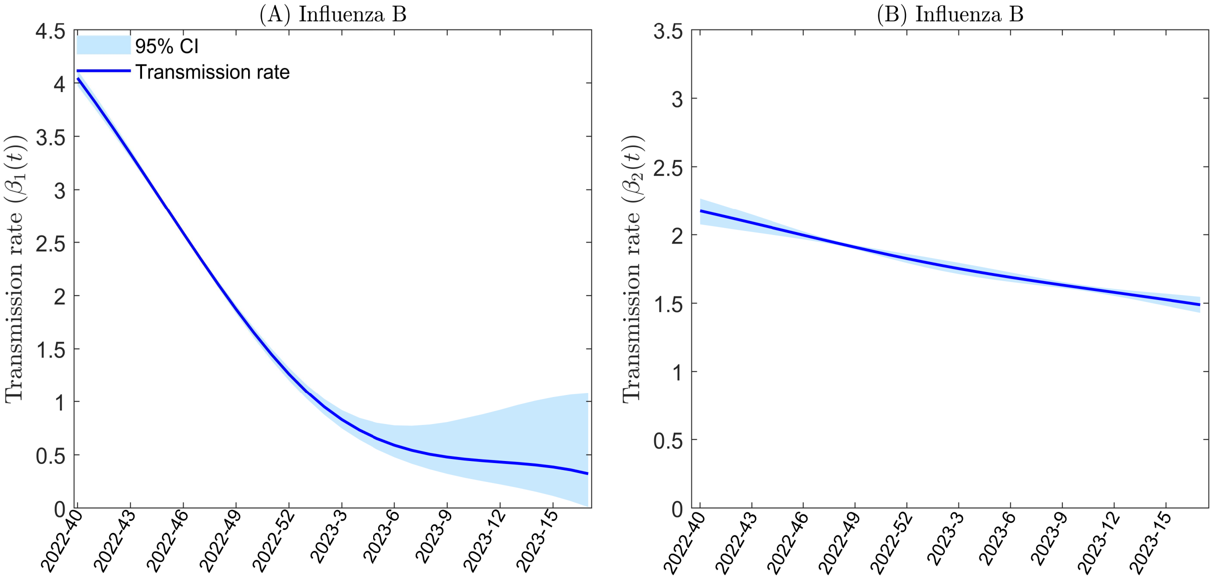

Fig S: Estimated time-varying transmission rates of influenza A and B in the United States (week 40 of 2022 to week 17 of 2023). Panels A and B show the modeled transmission rates for influenza A and B strains, respectively, derived from cubic spline functions ($n_\beta = 3$).

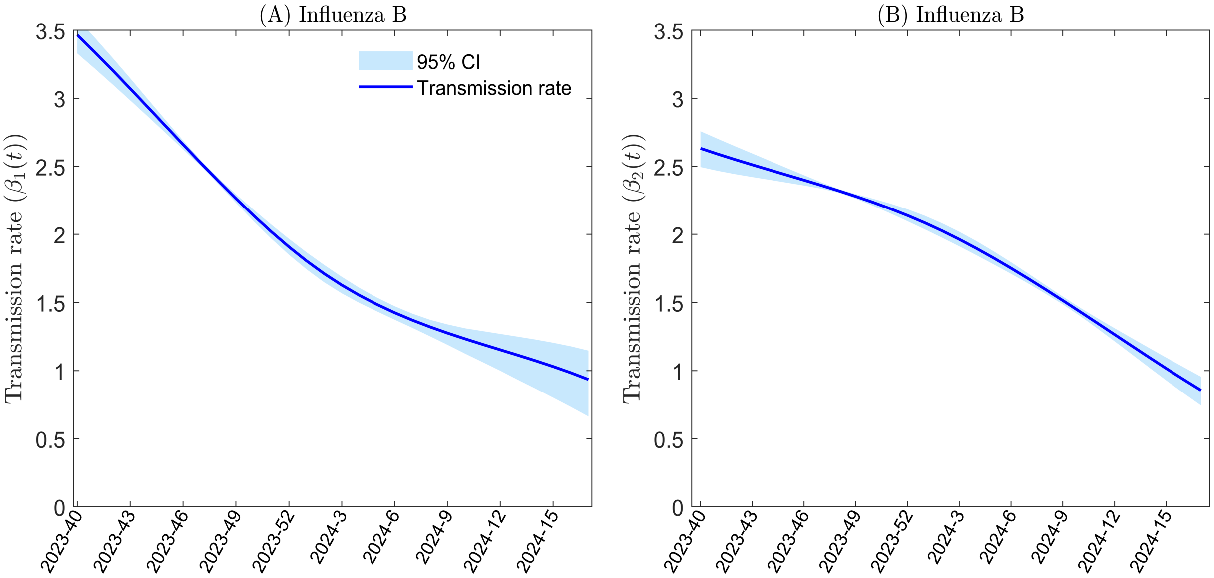

Fig T: Estimated time-varying transmission rates of influenza A and B in the United States (week 40 of 2023 to week 17 of 2024). Panels A and B show the modeled transmission rates for influenza A and B strains, respectively, derived from cubic spline functions ($n_\beta = 3$).

5 Simulation results of population susceptibility

In this section, we present simulations of the proportion of susceptible individuals to influenza in the United States using two distinct modeling frameworks: an age-structured model and a multi-strain model. For the age-structured model, the proportion of susceptible individuals within each age group (0-17, 18-64, and 65+ years) is calculated as $\frac{S_1}{N_1}$, $\frac{S_2}{N_2}$, and $\frac{S_3}{N_3}$, respectively, with results visualized across three panels (Panels A, B, and C of Fig U). For the multi-strain model, the proportion of susceptible individuals is derived as $\frac{S}{N}$, as shown in Fig V.

Our simulations suggest that the resurgence of pre-pandemic transmission levels appears attributable not to broad alterations in population immunity, but rather to the reestablishment of typical contact patterns following pandemic restrictions and ongoing viral evolutionary adaptations. While population susceptibility remained relatively stable, our modeling reveals subtle age-stratified variations in infection risk, particularly demonstrating marginally reduced susceptibility among young adults and adults in the post-pandemic period. These epidemiological patterns cannot be detected through traditional aggregated surveillance data, highlighting the critical value of computational modeling in uncovering hidden transmission dynamics within population subgroups.

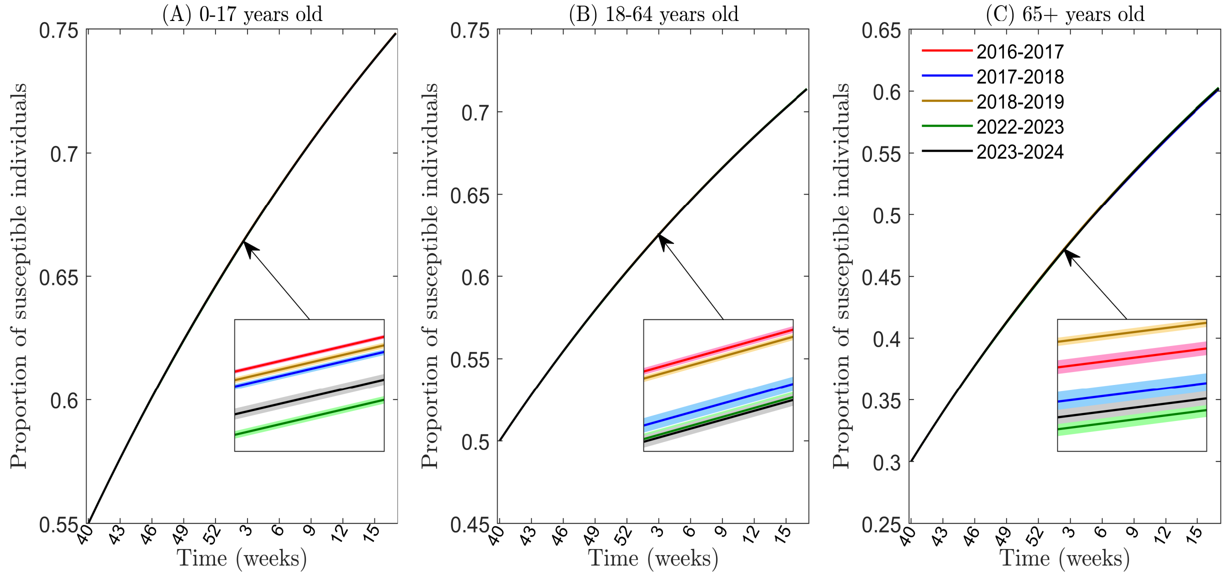

Fig U: Estimation of the proportion of individuals susceptible to influenza among those aged 0-17, 18-64, and 65+ in the United States using an age-structured model. Panels A, B, and C show the proportion of susceptible individuals to influenza among individuals aged 0-17, 18-64, and 65+, respectively. The 95% CI (confidence interval) for the simulations conducted in the years 2016-2017, 2017-2018, 2018-2019, 2022-2023, and 2023-2024 are plotted in pink, light blue, light yellow, light green, and gray, respectively.

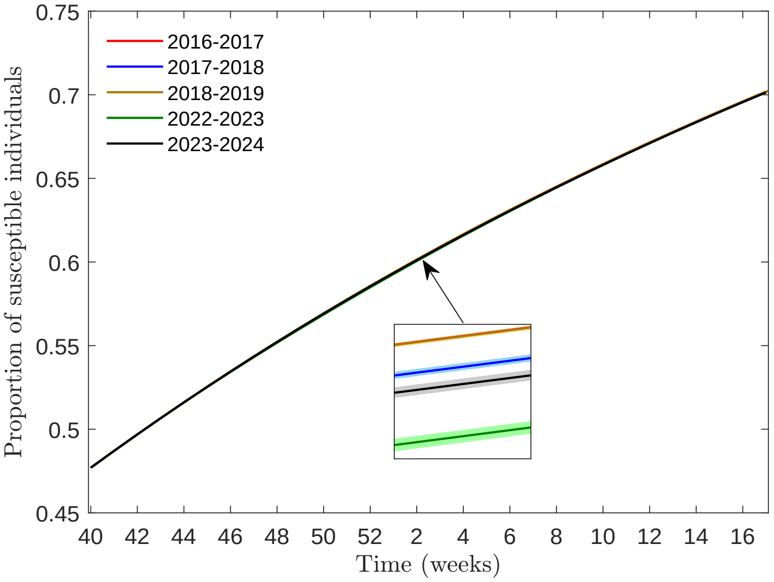

Fig V: Estimation of the proportion of individuals susceptible to influenza in the United States using a multi-strain model. The 95% CI for the simulations conducted in the years 2016-2017, 2017-2018, 2018-2019, 2022-2023, and 2023-2024 are plotted in pink, light blue, light yellow, light green, and gray, respectively.

References

[1] P. Van den Driessche, J. Watmough, Reproduction numbers and sub-threshold endemic equilibria for compartmental models of disease transmission, Mathematical Biosciences 180 (1-2) (2002) 29–48.

[2] O. Diekmann, J. A. P. Heesterbeek, J. A. Metz, On the definition and the computation of the basic reproduction ratio r0 in models for infectious diseases in heterogeneous populations, Journal of Mathematical Biology 28 (4) (1990) 365–382.

[3] World Health Organization. Population growth rate, United States of America, 2022; 2022. Available from: https://data.who.int/countries/840.

[4] World Health Organization. Influenza (seasonal); 2023. Available from: https://www.who.int/news-room/fact-sheets/detail/influenza-(seasonal).

[5] Jing S, Huo H, Xiang H. Modelling the effects of ozone concentration and pulse vaccination on seasonal influenza outbreaks in Gansu Province, China. Discrete and Continuous Dynamical Systems-B. 2022; 27 (4): 1877–1911. http://dx.doi.org/10.3934/dcdsb.2021113

[6] Centers for Disease Control and Prevention. Flu Vaccination Coverage, United States, 2022-2023 Influenza Season; 2024. Available from: https://www.cdc.gov/flu/fluvaxview/coverage-2223estimates.htm.

[7] Centers for Disease Control and Prevention. Laboratory-Confirmed Influenza Hospitalizations; 2024. Available from: https://gis.cdc.gov/grasp/fluview/FluHospChars.html.

[8] Our World in Data. Influenza; 2024. Available from: https://ourworldindata.org/influenza.

[9] Centers for Disease Control and Prevention. CDC Museum COVID-19 Timeline; 2024. Available from: https://www.cdc.gov/museum/timeline/covid19.html.

[10] World Health Organization. Statement on the fifteenth meeting of the IHR (2005) Emergency Committee on the COVID-19 pandemic; 2023. Available from: https://www.who.int/news/item/05-05-2023-statement-on-the-fifteenth-meeting-of-the-international-health-regulations-(2005)-emergency-committee-regarding-the-coronavirus-disease-(covid-19)-pandemic.
